# Online Chemical Analysis of Flowing *n*‑Hexane in a Pyrolysis Reactor by Optical Spectroscopy and Molecular Beam Mass Spectrometry

**DOI:** 10.1021/acs.jpca.6c00148

**Published:** 2026-03-09

**Authors:** Matthew C. Rohan, Cole J. VanDyke, Michael S. Hanchak, Elizabeth M. Craft, Elizabeth S. Kurian, Alexander D. Tucker, William K. Lewis, Andrew F. DeBlase

**Affiliations:** † 33319 Air Force Research Laboratory, Aerospace Systems Directorate, Wright-Patterson Air Force Base, Dayton, Ohio 45433, United States; ‡ 419111 University of Dayton Research Institute, Dayton, Ohio 45469, United States

## Abstract

Hydrocarbon pyrolysis at high pressures and temperatures is relevant to the decomposition of aviation fuel in advanced thermal management applications. To unravel the dynamics of hydrocarbon cracking and surface deposition, we have developed a novel experimental technique to characterize a neat, supercritical hydrocarbon fluid undergoing pyrolysis in a glass tube reactor (GTR). Using optical absorption spectroscopy, we sensitively measure the onset and rate of amorphous carbon deposition. Simultaneously, we unravel the chemical speciation of the fluid by online quadrupole mass spectrometry (MS). For *n*-hexane, we reveal four chemical regimes with increasing temperature: (1) no chemistry, (2) cracking with little-or-no deposition, (3) cracking with deposition, and (4) rapid, severe deposition. By modeling the GTR using computational fluid dynamics, we validate its representation as a simple plug flow reactor. The fluid phase decomposition of *n*-hexane, evident by MS, is consistent with an overall first-order process with an activation energy of 217.7 ± 2.4 kJ·mol^–1^. The temperature-dependent deposition rate is analyzed by a crude two-step model, and we compare our findings to those previously reported [e.g., Pramanik, M., et al. *Ind. Eng. Chem. Process Des. Dev.* **1985**, 24 (4), 1275–1281]. We anticipate that our experimental methods will provide a powerful means to quickly evaluate purported decomposition mechanisms of hydrocarbon fuel surrogates.

## Graphical abstract

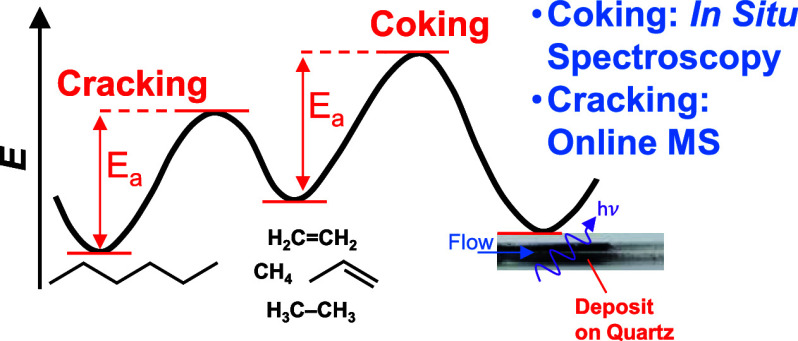

## 1 Introduction

Aviation fuel is the primary thermal management fluid onboard aircraft.^1–4^ In advanced propulsion applications, the hydrocarbon fuel may be subjected to temperatures (*T*) and pressures (*P*) in excess of its critical point (*T* ≥ 370 °C and *P* ≥ 2 MPa).^1^ At these conditions, it is essential that the fuel does not form destructive coke deposits that foul system components such as fuel injector nozzles, valves, filters, and other passages.^1,3^ Although initial cracking reactions with positive enthalpy may provide a beneficial heat sink, secondary reactions can occur that are often exothermic and generate coke precursors.^5,6^ Therefore, it is critical to mechanistically understand the relationships between decomposition products and the rates at which carbonaceous deposits accumulate. In the current study, we describe a novel experimental method that combines online molecular beam mass spectrometry (MS) with in situ optical absorption spectroscopy to probe the kinetics that link fluid phase pyrolysis products to surface deposits at high *T* (400–700 °C) and *P* (4.4 MPa).

At supercritical conditions, it is well-known that the fluid phase product distributions differ from those observed during gas phase pyrolysis.^1,7^ For example, while unimolecular β-scission reactions are believed to dominate gas phase chemistry, bimolecular H atom abstraction reactions are more likely in the denser supercritical environment, leading to paraffin rather than olefin primary products. Although beneficial mechanistic insights have been gained by studying the gas phase decomposition of relevant hydrocarbons in both flowtube^8,9^ and shocktube^10,11^ reactors, any gas phase models may require refinement when applied to supercritical fluids. Furthermore, the distributions of secondary pyrolysis products have been challenging to model, which led Ward et al.^12,13^ to exclude secondary products when developing the proportional product distribution (PPD) model for normal­(*n*)-alkanes. The PPD model assumes a single Arrhenius step for cracking the *n*-alkane into a primary product slate of fixed stoichiometry. More recently, this mechanism was expanded by Jiang et al.^14^ to include numerous secondary reactions, albeit with few routes to aromatic products (e.g., benzene, toluene, and xylene). Before applying such models to design thermal management systems, further validation is necessary with fuel surrogate compounds (e.g., *n*-alkanes) at conditions where the reaction times (*t*), *T*, and *P* are well controlled. Moreover, experimentally measured product distributions should reflect the in situ composition of the fluid in the reactor rather than the ex situ composition after the fluid is cooled to ambient *T* for offline analysis to avoid unwanted follow-on chemistry (e.g., asphaltic coke or condensation to tar).^1,15^

In our previous work, we employed molecular beam MS to sample hydrocarbon model compounds (e.g., *n*-hexane,^16–19^ cyclohexane,^16^ and *n*-dodecane^16,18,19^ ) and a supercritical logistical fuel (Jet A)^16^ to provide in situ chemical speciation. A schematic of our molecular beam MS apparatus is shown in Figure 1. According to this approach, the fluid at high pressure (3–7 MPa) is heated in a stainless-steel nozzle to 200–1000 °C. The neat fluid undergoes a supersonic expansion through the 10 μm nozzle into a vacuum chamber, where the expansion is skimmed to form a molecular beam. Because the molecules in the beam travel with a very narrow distribution of forward velocities, intermolecular collisions quickly diminish once the molecular beam is formed.^20,21^ Our methodology was inspired by a long line of work using supersonic expansions and molecular beams to trap and interrogate elusive gas phase species such as water clusters,^22,23^ metal complexes,^24,25^ biomolecules,^26,27^ and combustion intermediates.^28,29^ In our application, this methodology helps to eliminate any unwanted condensation chemistry that would otherwise be observed in the condensed phase as the fluid cools after pyrolysis. The molecular beam in our experiment is ionized by low energy (10–15 eV) electron-impact (EI) ionization to minimize fragmentation. The mass spectrum is then measured using a quadrupole mass filter. Using this technique, we obtain real time “snapshots” of the fluid speciation as the reactor *T* is varied. In the present work, we apply online MS to probe the fluid speciation in a flowing reactor while simultaneously measuring the in situ deposition rate by optical spectroscopy.

**Figure 1:**
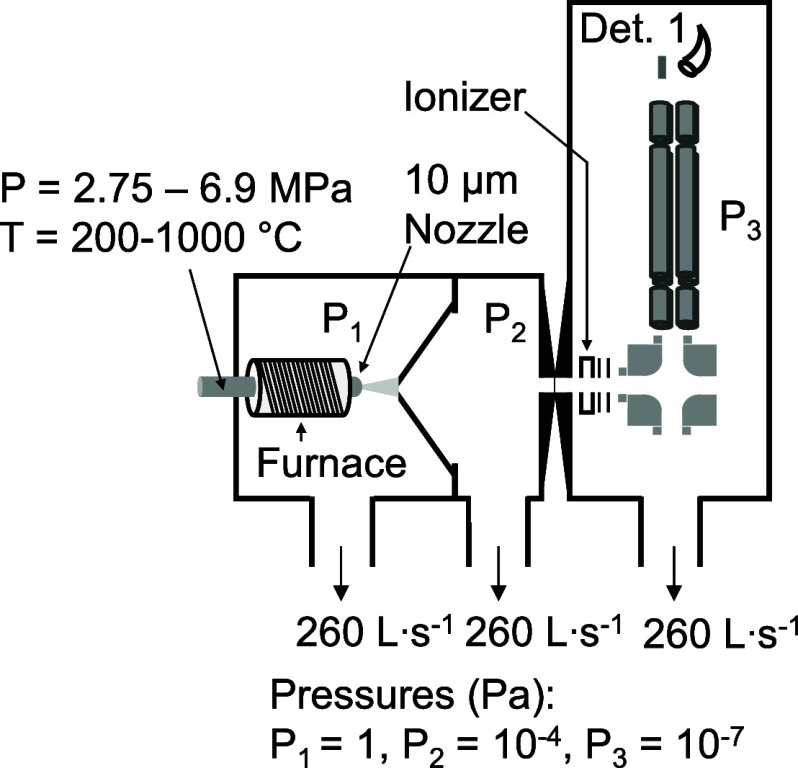
Diagram of the molecular beam MS instrument at Wright-Patterson Air Force Base for probing the chemical composition of supercritical hydrocarbon fluids.

To date, relatively few in situ methods have been demonstrated for measurement of the deposit accumulation that are suitable to pyrolysis conditions. Although Zabarnick and co-workers^30,31^ have developed a quartz crystal microbalance (QCM) method sensitive to autoxidative deposits, this device typically requires liquid samples and is not rated to withstand the high *T* and *P* conditions of interest here. Recently, Heldens and co-workers^32^ tested both thermal conductivity and speed of sound transducers for real time deposition measurements applied to catalytic methane pyrolysis. However, these tests were limited to gas phase conditions (0.2 MPa) and were highly sensitive to the implementation of the techniques.

In the current study, we apply our in situ optical spectroscopy method^33^ to measure deposition rates. This method is minimally invasive to the reactor, which is a transparent quartz tube. In addition to enabling optical access, the quartz reactor allows us to examine deposition chemistry on a relatively inert surface so that we can reduce the complexity of the problem in our initial work. In contrast, metal surface sites are known to catalyze the growth of filamentous coke, which blends with the amorphous and/or graphitic carbon deposits that originate from solution phase precursors.^1,3^ Herein, we combine in situ optical spectroscopy in a quartz tube reactor with online MS to link the abundance of fluid products to the rate of amorphous carbon deposition. This strategy has utility for validating coking models applicable to hydrocarbon fuel surrogates at idealized conditions, where *P*, *T*, and *t* are well-defined and catalytic surface reactions are likely negligible.

There have been multiple attempts to model pyrolytic deposition, which range from fully empirical^34^ to phenomenological^35^ approaches. In 1985, Kunzru and co-workers^34^ proposed a simple two-step empirical deposition model for gas phase *n*-hexane, which included conversion of the reactant into cracking products followed by the synthesis of coke from ethylene. In recent years, both empirical models and numerical methods have been applied to compute deposition rates at conditions relevant to propulsion applications, which have been reviewed.^15,36^ For example, Liu and co-workers^37^ have developed the empirical MC-II model, which distinguishes between catalytic coking on metal surfaces and lateral coking on previously coked surfaces. Based on their studies of *n*-decane and Chinese No. 3 jet fuel, they found that the concentrations of propylene and aromatics (total concentration of benzene, toluene, and *p*-xylene) could be related to the rates of catalytic and lateral coking, respectively. In addition, Wang and co-workers^35^ have developed a coking random pore model (C-RPM) to describe the growth and fusion of coke particles in a mechanism analogous to char gasification. In all these studies, coke was deposited on metal surfaces, necessitating the full treatment of catalytic vs. lateral growth mechanisms during model development. Furthermore, the masses of deposits on test articles were measured ex situ, either by gravimetric^34^ or carbon burnoff^37^ methods. Consequently, the previous experimental methods would not capture dynamic changes in deposition rates throughout a test.

In this work, we demonstrate in situ optical spectroscopy and online MS as powerful model validation tools for a simple reactor geometry with a noninteracting surface, a quartz tube. Using this glass tube reactor (GTR), we show that distinct regimes of pyrolysis chemistry can be easily identified in the fluid phase as a function of *T*. We relate these regimes to their corresponding in situ deposition rates on the quartz surface. Presently, we focus on *n*-hexane because its gas phase decomposition chemistry has been well-studied,^34,38–40^ which is useful for validation purposes. Finally, we generate Arrhenius plots from our data to evaluate the relationships between common fluid phase products and the measured deposition rates.

## 2 Experimental Methods

### 2.1 Materials

The *n*-hexane was 99+% pure and was purchased from Fisher Scientific. To eliminate the effects of autoxidative deposition,^41,42^ 260 mL of the fluid was sparged for several hours with N_2_ before each experiment.

For the GTR, quartz tubes were purchased from Quartz Scientific Inc. and had outer diameters (o.d.) of 6 mm and inner diameters (i.d.) of either 1 mm (100001DD48) or 4 mm (100004B48). The tubes were cut into 70 cm segments to be used as test articles.

### 2.2 Instrumentation

#### 2.2.1 The GTR

A schematic of the GTR for in situ optical spectroscopy is given in Figure 2. A syringe pump (ISCO 260D) is used to provide a constant volumetric flow of *n*-hexane through the rig. The GTR is composed of three furnaces, a heated transfer line with a 0.5 μm inline filter (Swagelok, SS-2F-05), a spill return sampling nozzle, and a back pressure regulator. Before flowing hydrocarbon fluids through the system, the lines are flushed with N_2_ using the bypass valve to prevent autoignition. The emergency vent valve is used to relieve pressure in the event of an obstruction inside the reactor.

**Figure 2:**
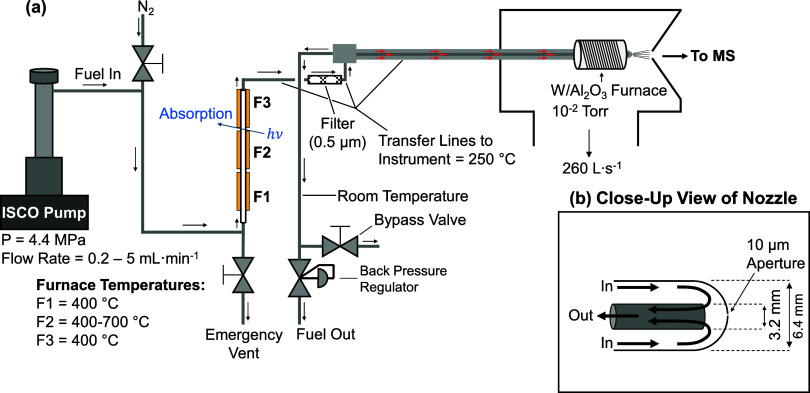
Diagram of the GTR. The flow path and typical fluid conditions are given in (a), while an expansion of the supersonic expansion nozzle is shown in (b). Note that the mass spectrometer is the same as depicted in Figure 1.

The reactor comprises three furnaces (F1, F2, and F3 in order of flow), which are vertically oriented to minimize buoyancy effects. Each furnace is a copper tube (19 mm o.d., 6.4 mm i.d., and 15.2 cm long) wrapped with heating ribbon (McMaster Carr, 120 VAC, 156 W, 61 cm long, rated to 760 °C). The heaters are insulated using ceramic fiber insulation (Morgan Cerablanket). The quartz tube test article (6 mm o.d.) is inserted through the i.d. of each of the three furnaces, which are separated by stainless steel bushings (6.4 mm thick, 6.4 mm i.d., and 12.7 mm o.d.). The *T* values of the copper furnaces are measured using K-type thermocouple probes, which are inserted into 1.6 mm holes in the copper to ensure stable contact. Two thermocouples are used for each furnace, where one is connected to a proportional-integral-derivative (PID) controller (BriskHeat) and the other is connected to a National Instruments data acquisition module (NI9211) for real time *T* measurements. To reach the highest *T* settings in F2, a step-up transformer (Variac) is used to increase the PID output to 140 VAC.

During typical operation of the GTR, F1 preheats the fluid so that its temperature (*T*_F1_) is above the critical point but below pyrolysis onset, *T*_F2_ is the reactor temperature varied in the experiment, and *T*_F3_ is again above the critical point but below the temperature for pyrolysis. For experiments with *n*-hexane, *T*_F1_, *T*_F2_, and *T*_F3_ values were chosen so that a phase change between subcritical and supercritical did not occur near the pyrolysis reactor (F2). Because the critical *T* of *n*-hexane is 234.4 °C,^43^ *T*_F1_ and *T*_F3_ were both set to 400 °C, which was well below any pyrolysis *T* observed in this work. The values of *T*_F2_ were varied and set as high as 700 °C to drive pyrolysis chemistry in this study. After exiting F3, the fluid passes through heated transfer lines into the source chamber of the mass spectrometer. These lines are set to 250 °C using additional PID controllers. For the current design at an inlet flow rate of 0.2 mL·min^–1^, it takes up to 25 min for *n*-hexane to reach the mass spectrometer from the exit of F3. We expect this time to decrease as the conversion increases and more mobile gaseous products are generated. Therefore, the dwell time at each *T*_F2_ condition was 30 min to allow ample time for the product distribution to equilibrate. After exiting the mass spectrometer, the fluid returns to ambient *T* and exits the rig through a back pressure regulator. Throughout this investigation, the back pressure regulator (EquiliBAR ZF1SNN8) was set to 4.4 MPa, which is well above the critical pressure of *n*-hexane (3.02 MPa).^43^ During the experiments, the ISCO pump was run with a constant flow rate of 0.2 mL·min^–1^.

#### 2.2.2 Mass Spectrometry

Our molecular beam mass spectrometer is based on the Extrel/Process Insights VeraSpec system and has been described previously.^16,17^ This instrument has been interfaced with the GTR to provide real time measurements of fluid speciation. Using a spill return nozzle design (Figure 2b), continuously flowing fuel is sampled by the mass spectrometer through a 10 μm aperture. If this aperture becomes occluded or clogs, the residence time (*t*) in the nozzle should remain constant despite the reduced MS signal. In our previous work,^16,17^ *t* increased with *T* as deposits formed on the nozzle and the flow into the mass spectrometer decreased. The new design allows *T* and *t* to be decoupled, which is well-suited for kinetics measurements. The spill return nozzle is composed of a 3.2 mm o.d. tube inserted into a 6.4 mm o.d. nozzle that terminates in a laser-drilled 10 μm aperture (Lenox Laser). The fluid flows toward the aperture around the outside of the 3.2 mm o.d. tube and exits through the inside of this tube so that the fluid T entering the aperture is as close as possible to the furnace set point *T*. The furnace surrounding the nozzle is custom built and consists of a 2.54 cm long Al_2_O_3_ tube (Accuratus: 9.5 mm o.d. and 6.4 mm i.d.) wound with a 0.3 mm diameter tungsten wire (McMaster Carr). By applying 6 VDC to this wire, the *T* is maintained at about 380 °C. In contrast to our previous work,^16,17^ pyrolysis chemistry occurs in the GTR rather than within the supersonic expansion nozzle. In the current study, the nozzle is heated to ensure that the fluid is maintained above its critical *T* but below the pyrolysis threshold during sampling.

#### 2.2.3 Optical Spectroscopy

Optical absorption spectroscopy is performed 2.5 cm below the top of F2 to ensure that the fluid is in thermal equilibrium with the furnace wall where the transmission is measured. An LED light source (ThorLabs, SOLIS-405D), is fiber-coupled to an optical pitch and catch on both sides of F2. The light transmitted through the quartz tube between the pitch and catch is collimated using Ocean Optics lenses (74-UV) mounted on both the pitch and the catch fibers. These lenses screw into custom machined ceramic standoff pieces (Macor, 5 cm long) that connect them to the copper furnace to provide thermal insulation and avoid damage to the lenses. Light from the optical catch passes through a 650 nm short pass filter (ThorLabs, FESH0650) to remove any contributions from blackbody radiation of the furnace.

The intensity of the filtered light is measured using a silicon photodiode detector (ThorLabs, Det100A2). The signal from this detector is measured by a National Instrument analogue input cDAQ module (NI9215), which is terminated through a 1 kΩ resistor. A separate detector is used to measure the intensity of light directly from the LED source to verify its stability throughout each experiment. A LabView program is used to record these signals as well as the furnace *T* values throughout the experiment. A separate program written in Python synchronizes the time axes of the optical and MS experiments.

To determine the best wavelength for measuring the transmitted light (405 nm), the UV/vis spectrum of the deposit and fluid was obtained using an Avantes spectrometer (AvaSpec-HSC1024x58) with a halogen lamp light source from Thor Laboratories (OSL2). To measure the spectrum of deposits, spent tubes were removed from the GTR, drained, dried, and then inserted into a separate fiber-coupled sample holder. The spectrum of the fluid was measured in a standard 1 cm quartz cuvette.

To measure the average surface density (σ) of carbon in μg·cm^–2^, a calibration plot was constructed that relates the optical absorbance (*A*) of a deposit to σ. The deposit A was measured in 1 cm increments along quartz tubes from two runs by removing these tubes at the end of the runs and inserting them into a separate fiber-coupled jig. These 1 cm increments were cut into sections that were burned off to measure the carbon mass using a LECO instrument. Note that the size of the columnated light beam is about 1 cm in diameter so that the absorbance should be proportional to the average surface density of carbon in each section according to the Beer–Lambert Law. For these runs, *n*-hexane was flowed through a 4 mm i.d. quartz tube at 0.2 mL·min^–1^ with *T*_F1_ = *T*_F3_ = 400 °C and *T*_F2_ = 650 °C. To accumulate enough deposit for the carbon burnoff calibration, one run lasted 80 min, while the other lasted 180 min.

### 2.3 Modeling and Simulations

#### 2.3.1 Computational Fluid Dynamics

To better understand the flow behavior in the quartz tube reactor, two-dimensional computational fluid dynamics (2D-CFD) simulations were performed using the Ansys Fluent software.^44^ The GTR was assumed to be axisymmetric with a total length of 575 mm. Both 1 and 4 mm i.d. quartz tubes were simulated. The wall thickness of the tube was included in the simulation. The o.d. of the quartz tube was assumed to be in intimate contact with the copper or stainless-steel surfaces.

The computational mesh of the fluid domain was divided into 0.1 mm length axial cells. The radial direction was partitioned into 20 cells with a 4× bias factor to create smaller cells near the wall to more accurately capture the transport and heat transfer. A mesh refinement study ensured that a fine enough discretization was achieved.

The inlet to the reactor was modeled as a velocity boundary condition (BC) based on the volumetric flow rate and inlet cross-sectional area. Consistent with the low flow rate (0.2 mL·min^–1^), the Reynolds numbers were calculated to be much less than 1000, especially near the center of F2. Therefore, laminar flow was assumed.

In the first 80.4 mm of the rig, the outer diameter of the tube was subjected to a convection BC of 10 W·m^–2^·°C^–1^ at 22 °C. The values of *T*_F1_, *T*_F2_, and *T*_F3_ were set for the copper parts to represent the experimental conditions, and axial heat transfer between the furnaces through the stainless steel spacers was explicitly modeled (relevant dimensions in Section 2.2.1). Fluid properties of *n*-hexane were calculated with the Redlich–Kwong equation of state and temperature-dependent curve fits.

#### 2.3.2 Equivalent Volume Approximation

To define the reaction time, *t*, we examined both the time at which the fluid is approximately isothermal at *T*_F2_ and the time during which the fluid increases in *T* near the beginning of F2. As *T* increases when the fluid traverses F2, the rate constants (*k*) should also increase. To account for this effect, we applied the equivalent volume approximation.^45^ According to this ansatz, a reaction with the rate *r*_A_ in mol·L^–1^·s^–1^ in a reactor with a volume of *V* is related to the same reaction with the rate *r*_AE_ in an equivalent volume (*V*_E_) by the differential relationship:

$$r_{A}dV=r_{\mathrm{AE}}dV_{E}$$ 1

To evaluate *V*_E_, we assume Arrhenius rate laws for *r*_A_ and *r*_AE_ such that

$$r_{A}=k_{0}\exp\left(-\frac{E_{a}}{RT}\right)[A{]}^{n}=\frac{k_{0}}{M^{n}}\exp\left(-\frac{E_{a}}{RT}\right)\rho_{z}^{n}$$ 2

and

$$r_{\mathrm{AE}}=k_{0}\exp\left(-\frac{E_{a}}{RT_{E}}\right)[A{]}_{E}^{n}=\frac{k_{0}}{M^{n}}\exp\left(-\frac{E_{a}}{RT_{E}}\right)\rho_{E}^{n}$$ 3

where *k*_0_ is the Arrhenius constant in units of L*^n^* ^–1^·mol^1‑*n*^·s^–1^, *M* is the molar mass in g·mol^–1^, *E*_a_ is the activation energy in J·mol^–1^, *R* is the ideal gas constant in J·mol^–1^·K^–1^, *T* is in K, [*A*] is the molar concentration of a reactant A, *n* is the order of the reaction, and ρ_z_ is the mass density of the fluid along the flow axis of the reactor (z) in kg·m^–3^. Substituting ([Disp-formula eq2]) and ([Disp-formula eq3]) into ([Disp-formula eq1]), rearranging, and integrating to find *V*_E_ we have

$$V_{E}=\int_{0}^{V}{\left(\frac{\rho_{z}}{\rho_{E}}\right)}^{n}\exp\left[-\frac{E_{a}}{R}\left(\frac{1}{T}-\frac{1}{T_{E}}\right)\right]dV$$ 4

Using the values of ρ_*z*_ and *T* from the CFD calculation, ([Disp-formula eq4]) can be numerically integrated after defining ρ_E_, *T*_E_, and *E*_a_. In this study, *T*_E_ was set equal to the F2 set point *T* and ρ_E_ was set equal to the density of *n*-hexane corresponding to *T*_E_ according to the equation of state. We used a value of *E*_a_ = 208.5 kJ·mol^–1^ based on the thermal decomposition of gas phase *n*-hexane in a plug flow reactor.^46^ The value calculated for *V*_E_ can be used to define the equivalent reaction time (*t*_E_) as follows:

$$t_{E}=\frac{V_{E}}{v_{E}}=\frac{V_{E}\rho_{E}}{v_{0}\rho_{0}}$$ 5

where *v*_E_ is the volumetric flow rate at *T*_E_, *v*_0_ is the inlet volumetric flow (25 °C), and ρ_0_ is the inlet fluid density (25 °C and 4.4 MPa).

Densities must be included in ([Disp-formula eq5]) to satisfy conservation of mass within the reactor. After the liquid fluid undergoes the supercritical phase change, its density rapidly decreases so that less mass (*V*_E_ρ_E_) can exist within the reactor at higher *T* than the mass that could fill the same volume at subcritical conditions. Because the volumetric flow rate at the pump (*v*_0_) is defined at room temperature, we divide by *v*_0_ρ_0_ in ([Disp-formula eq5]) to preserve the mass balance. Note that *t*_E_ should decrease with increasing *T*_E_ because ρ_E_ decreases for a real gas.

To calculate *V*_E_, we assumed that the fluid density is not influenced by the densities of the product species. This assumption is well justified at low conversion for identifying the initial onset *T* of decomposition. Furthermore, the densities of abundant products (e.g., methane and ethylene) are close to the density of *n*-hexane at high *T*. For example, the density of *n*-hexane at 700 °C and 4.4 MPa is 0.553 mol·L^–1^ while the corresponding densities of methane and ethylene are 0.534 and 0.535 mol·L^–1^, respectively, according to NIST SUPERTRAPP.^47^

## 3 Results and Analysis

### 3.1 Wavelength Selection for Optical Spectroscopy

To quantify the amount of deposit, we applied optical spectroscopy assuming the Beer–Lambert Law ([Disp-formula eq6]), where *A* is absorbance, α­(λ) is the molar absorptivity at a wavelength (λ), *b* is the path length in cm, and *c* is the molar concentration. To measure σ for the deposit, we define the proportionality constant β­(λ) according to ([Disp-formula eq6]), where *M* is the molar mass of the deposit in g·mol^–1^. Recall from above that σ is the average surface density of carbon in μg·cm^–2^. The linear relationship between σ and *A* should hold, assuming that the sample is thin, homogeneous, free from particles that scatter light, and not optically saturated.

A=α(λ)bc=β(λ)σ 6

$$\beta (\lambda )=10^{-3}\;M^{-1}\alpha (\lambda )$$ 7

To select the appropriate λ to apply the Beer–Lambert Law, we obtained spectra for both the deposit and thermally stressed *n*-hexane fluid (Figure 3). The spectra of the deposits at different *T*_F2_ are similar in shape and very broad throughout the range measured. The thermally stressed fluid was obtained from the waste container of the GTR after multiple runs and after partial evaporation to improve both the visibility of the color and signal-to-noise ratio of the spectrum. Note that for a single experiment, the color change is only barely visible at *T*_F2_ > 680 °C (Supporting Information, Section 1 and Figure S1). Consistent with its color, the UV/vis spectrum of the fluid is less broad than the deposit and absorbs most strongly at the shorter wavelengths. The absorbance of both the deposit and fluid increases as λ decreases throughout the visible region; therefore, we chose the shortest practical λ at which an LED lamp could be purchased (405 nm). We did not select a UV wavelength to avoid any potential complications from photolysis. With an optical absorption method in hand to measure the deposition in F2, we proceeded to combine this technique with online MS to simultaneously characterize the fluid speciation.

**Figure 3:**
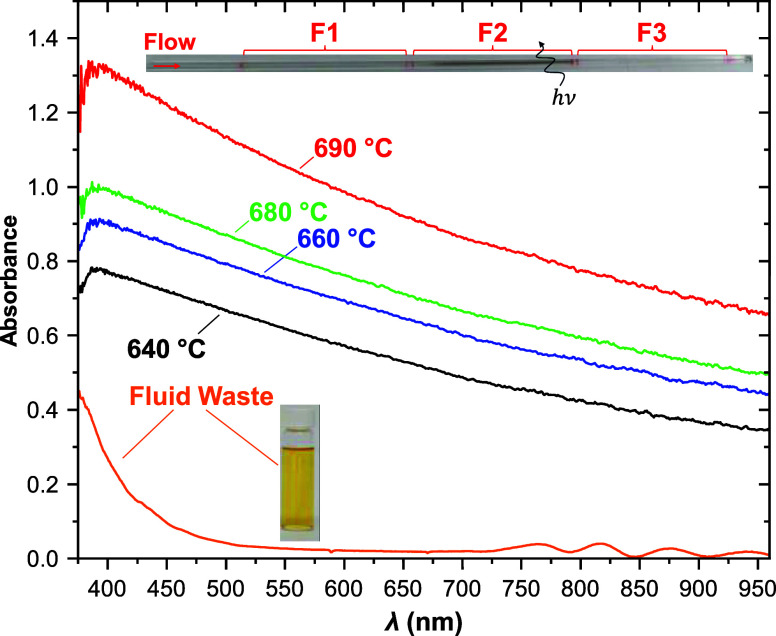
Optical Spectra of carbon deposits formed at different *T*_F2_ conditions (640 °C = black, 660 °C = blue, 680 °C = green, and 690 °C = red). In all experiments *T*_F1_ = *T*_F3_ = 400 °C. A photograph of a spent 1 mm i.d. quartz tube is at the top (conditions: F1 = 400 °C, F2 = 690 °C, and F3 = 400 °C), which shows a carbon deposit in the pyrolysis zone (F2) and indicates the location where the spectra were obtained. The spectrum of concentrated fluid waste from the GTR is given in the bottom trace with a photograph of the thermally stressed fluid.

### 3.2 Online MS and Absorption Spectroscopy in the GTR

To obtain real time chemical speciation data, the molecular beam MS instrument was integrated with the GTR. This apparatus allowed us to continuously sample a single-phase supercritical fluid as *T* varied in F2. The new design leverages insights from our previous work,^16–19^ in which pyrolysis occurred within the supersonic expansion nozzle to minimize condensation chemistry^1,15^ by preventing condensed phase cooling prior to sampling.

Given that *T* decreases from *T*_F2_ to 250 °C before sampling, we examine the possibility that condensation coking occurs. First, we note that deposition in F3 or in the transfer lines was minimal for most conditions studied, except for cases in which *T*_F2_ was maintained at the most extreme temperatures (e.g., 700 °C for a flow rate of 0.2 mL·min^–1^ in the 1 mm i.d. reactor) for at least 30 min. In these extreme cases, one may expect the MS product distribution to favor condensation products [e.g., polycyclic aromatic hydrocarbons (PAHs)] rather than reflecting the *in situ* composition of the fluid in the pyrolysis reactor (F2). This possibility is explored in the Supporting Information (Section 2) for an *n*-alkane surrogate, *n*-dodecane, which was pyrolyzed (1) within the supersonic expansion nozzle and (2) upstream of the supersonic expansion prior to the 250 °C heated transfer lines using a stainless-steel tube reactor (Figure S2). Note that we previously observed that *n*-hexane, *n*-dodecane, and other saturated hydrocarbons have common aromatic product distributions when heated to the most extreme temperatures (Figure S3). We observed that at 760 °C the mass spectra did not greatly differ for configurations (1) and (2) (Figure S4). This result, which applies to more extreme conditions than those studied herein, suggests that the presence of condensation coking is unlikely to substantially alter the composition of the fluid phase as measured by online MS. Therefore, we proceed to relate the speciation observed by online MS to the rate of deposition measured by absorption spectroscopy.

Representative mass spectra from a *T* ramp experiment in F2 are presented in Figure 4. Unless otherwise stated, the i.d. of the quartz reactor tube is 1 mm, *P* = 4.4 MPa and *T*_F1_ = *T*_F3_ = 400 °C throughout the manuscript. At *T* ≤ 550 °C, the spectrum corresponds to the 15 eV mass spectrum of *n*-hexane, which contains substantial fragmentation of the molecular ion (M^+^) into two- to five-carbon (C2–C5) species. Between 550 and 620 °C, the distribution appears to shift in favor of lighter alkyl cracking products (e.g., CH_4_^+^, C_2_H_4_^+^, C_2_H_6_^+^, and C_3_H_6_^+^), which become more pronounced at 660 °C. At 660 °C, aromatic species appear in the mass spectrum (e.g., benzene, toluene, and xylene/ethylbenzene), which grow in abundance at 700 °C.

**Figure 4:**
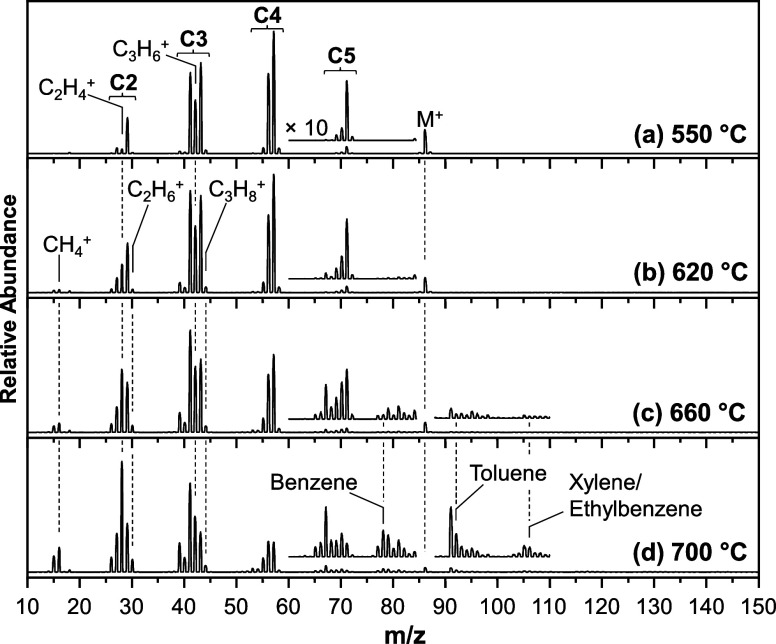
Mass Spectra of thermally stressed *n*-hexane in the GTR with a 1 mm quartz tube at 4.4 MPa and an inlet flow rate of 0.2 mL·min^–1^ for the following *T*_F2_ values: 550 °C (a), 620 °C (b), 660 °C (c), and 700 °C (d). Note that *T*_F1_ = *T*_F3_ = 400 °C. Relevant product species are labeled with guidelines and the spectra are expanded ×10 to show less abundant species.

In this work, we only use MS intensities to indicate that a species or a fragment is present and to track its abundance relative to the same species at an earlier time in the experiment. The complexity of the MS data is clearly exacerbated by the fact that EI fragment ions may overlap M^+^ ions of fluid phase product species. This complication is most evident when examining alkyl cracking products that are lighter than C_6_H_14_^+^. Several peaks (e.g., C_2_H_4_^+^ and C_2_H_6_^+^) grow in between fragment peaks, making these thermal products easier to distinguish. On the other hand, some species (e.g., C_3_H_6_^+^) likely correspond to both thermal products and EI fragments because they are present in the initial EI spectrum of *n*-hexane but do not exhibit a decrease in relative abundance that is proportional to the decrease in the M^+^ signal. This behavior indicates the production of isobaric species by thermal cracking in F2. In our previous work,^18^ we distinguished similar thermal cracking vs. EI fragments using a triple quadrupole mass spectrometer in precursor scan mode. Herein, we show only results from the single quadrupole instrument because these measurements are faster to pair in real time with the optical absorbance technique and provide a detailed qualitative picture of the fluid phase chemistry.

For some anticipated M^+^ ions of thermal products, substantial EI fragmentation into lighter ions is expected. Therefore, the relative fractional abundances (*x*_fa_) of peaks corresponding to M^+^ ions of product species should not be interpreted as the relative concentrations of different species. Furthermore, *x*_fa_ at 15 eV may differ based on the EI cross sections of the different species in addition to their concentrations.

In Figure 5, we plot both the in situ optical absorbance measurements and *x*_fa_ values of key ions monitored by online MS for a *T* ramp experiment in a 1 mm quartz tube reactor. Note that the key ions selected for Figure 5 are chosen to represent typical behaviors of product species throughout the *T* ramp and these ions are not meant to encompass all product and fragment species that are apparent in Figure 4. Results for the 4 mm i.d. reactor at the same conditions are presented in the Supporting Information (Section 3.1, Figure S5).

**Figure 5:**
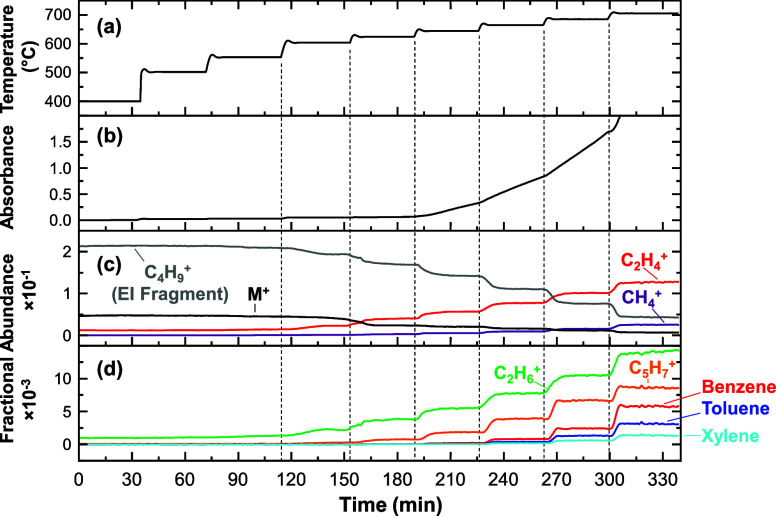
Experimental results from the GTR with a 1 mm i.d. quartz tube reactor at 4.4 MPa and an inlet flow rate of 0.2 mL·min^–1^. The wall temperature of F2 is plotted in (a) as a function of experiment time. The absorbance of 405 nm light through the quartz tube is given in (b). The fractional abundances of key species measured by MS are color coded and plotted in (c) and (d). The ordinate axis is expanded × 100 in (d) relative to (c) to better visualize minor ions.

To illustrate reproducibility of data from the GTR, the MS results from two duplicate survey scans with 4 mm i.d. reactors are overlaid in the Supporting Information (Section 3.2, Figure S6). The fractional abundances are quite stable after the GTR equilibrates to a given *T*_F2_, and can be effectively averaged over intervals of at least 5 min. Therefore, the main source of error between runs is likely attributed to the reproducibility of GTR conditions (e.g., the precise value of *T*_F2_). Based on Figure S6, we believe this variation is reasonable and should not affect the semiquantitative results in this study.

Focusing on the 1 mm i.d. reactor data in Figure 5, four distinct chemical regimes are apparent:

(1) No chemistry: *T* ≤ 550 °C.
(2) Cracking with little-or-no deposition: 600–620 °C.
(3) Cracking and deposition: 640–80 °C.
(4) Rapid, severe deposition: *T* ≥ 700 °C.

Regime (1) applies to *T* ≤ 550 °C and is marked by a negligible rate of increase in absorbance and also little-to-no change in the relative abundances of ions in the mass spectra.

In regime (2) (600 and 620 °C), the abundances increase for lighter alkyl products, which is evident by the increase in signal for the C_2_H_4_^+^, C_2_H_6_^+^, and C_5_H_7_^+^ ions. Concurrently, the fractional abundances of the M^+^ ion and the most intense EI fragment from the initial mass spectrum (C_4_H_9_^+^) decrease. Although cracking chemistry is now observed by MS, little change occurs in the absorbance in each *T* segment.

Unlike regime (2), in regime (3) (640–680 °C) a steady increase in the absorbance signal is observed. The slope of this absorbance change increases each time *T* is increased. Although this absorbance increase may be attributed to either the fluid or deposit, the contribution of the fluid to the absorbance should be constant if conditions (*T*, *P*, and *t*) are unchanged in the flow reactor. In contrast, the absorbance steadily increases as *T* is held constant, which is consistent with accumulation of deposit on the reactor wall. From the MS data, species associated with cracking products continue to increase in abundance and aromatic species (e.g., benzene, toluene, and xylene) begin to appear (*T* ≥ 660 °C).

In regime (4) (*T* ≥ 700 °C), there is a dramatic increase in the deposition rate so that the quartz tube becomes opaque within a few minutes. Product species, including aromatics, continue to increase in abundance in this regime. Regime (4) should likely be avoided in any thermal management system because of its highly destructive nature. Therefore, we focus the remainder of this study on understanding the progression between regimes (1)–(3) (*T* ≤ 680 °C) for a model compound, *n*-hexane, in an idealized reactor with a noninteracting quartz wall.

### 3.3 Modeling and Simulations

#### 3.3.1 CFD

To validate the apparatus, we fit the overall *E*_a_ for unimolecular decomposition of *n*-hexane in the GTR so that our result could be compared with existing literature.^7,46^ Figure 6 shows the fluid *T* profiles as *n*-hexane passes through the three furnaces of the 1 mm i.d. reactor (*P* = 4.4 MPa, *v*_0_ = 0.2 mL·min^–1^, *T*_F1_ = 400 °C, *T*_F2_ = 600 °C, and *T*_F3_ = 400 °C). Simulation results from the 4 mm i.d. reactor are given in the Supporting Information (Section 3.3, Figures S7 and S8). According to the simulations, the fluid is in excellent thermal equilibrium with the reactor wall well before the exit of F2.

**Figure 6:**
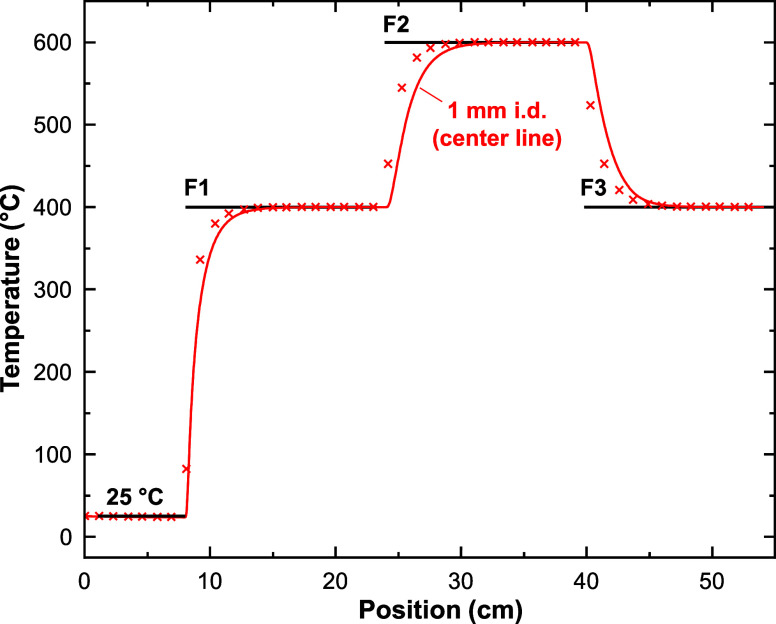
Temperature vs. position profile in the GTR calculated by 2D-CFD. The simulation was performed for *n*-hexane in a 1 mm i.d. reactor, which had an inlet flow rate of 0.2 mL·min^–1^ and *P* = 4.4 MPa. The black lines denote the *T* set points for the inlet fuel and furnaces. The solid red curve represents the centerline *T*. The red x’s indicate the mean radial *T* calculated at discrete positions along the reactor.

In general, the mean radial temperature (⟨*T*_r_⟩) of the fluid responds more quickly than the centerline temperature (*T*_c_) changes when moving from F1 to F2 to F3. This result is sensible because the fluid in the center of the tube should be the most insulated from the reactor wall, whereas ⟨*T*_r_⟩ includes contributions from *T*_c_ as well as the fluid near the wall.

To better visualize the relevant times for modeling chemistry in F2, Figure 7 illustrates both the warm-up time (*t*_warm‑up_) and the time at maximum *T* (*t*_Tmax_) for a series of CFD calculations that varied *T*_F2_. In this bar chart, *t*_Tmax_ is defined as the time that ⟨*T*_r_⟩ is within 1 °C of *T*_F2_ and *t*_F2_ = *t*_warm‑up_ + *t*_Tmax_, where *t*_F2_ is the residence time in F2. In Table 1, we compare values for *t*_F2_, *t*_Tmax_, and *t*_e_ (see Section 2.3.2 for calculation of *t*_e_). In all cases, *t*_Tmax_ < *t*_e_ < *t*_F2_. Note that *t*_e_ is not listed for *T*_F2_ = 400 °C because the equivalent *T*_F1_ and *T*_F3_ values make *t*_e_ artificially long and no reaction is observed at this low *T*. We proceed to use *t*_e_ as the reaction time in F2 over the temperatures of interest.

**Figure 7:**
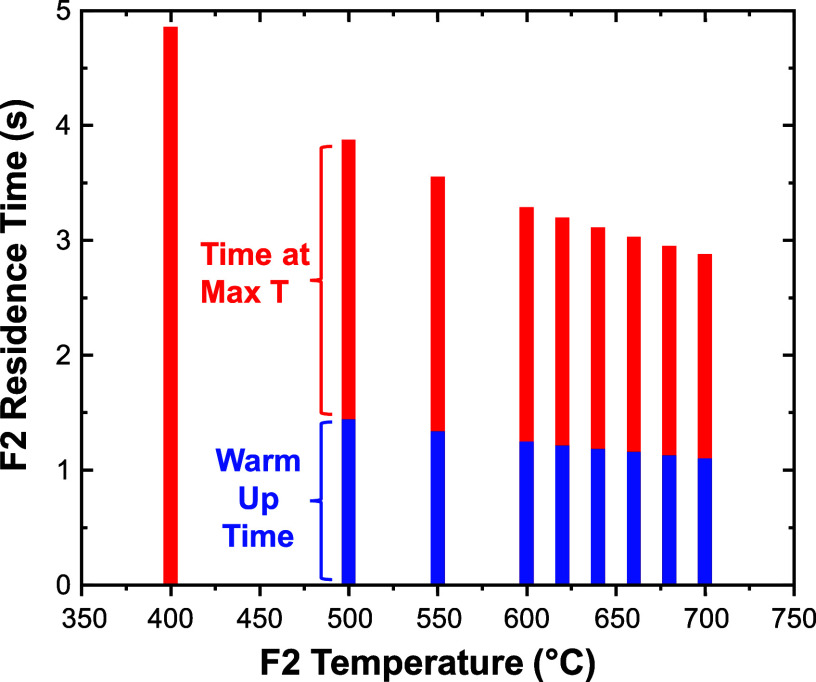
Total residence time in F2 (*t*_F2_) and its contributions from *t*_warm‑up_ (blue) and *t*_Tmax_ (red) for CFD simulations with different values of *T*_F2_.

**Table 1:** Values of *t*_F2_, *t*_Tmax_, and *t*_e_ Derived from 2D-CFD Simulations for *n*-Hexane in a 1 mm I.D. Quartz Tube Reactor

***T* (°C)**	***t***_**F2**_ **(s)**	***t***_**Tmax**_ **(s)**	***t***_**e**_ **(s)**
400	4.85	4.85	
500	3.87	2.43	3.38
550	3.55	2.22	2.94
600	3.28	2.04	2.67
620	3.19	1.98	2.58
640	3.11	1.93	2.50
660	3.03	1.87	2.43
680	2.95	1.82	2.37
700	2.88	1.78	2.31

#### 3.3.2 Fluid Decomposition of *n*-Hexane

To model the fluid decomposition of *n*-hexane, we applied a simple first-order lumped kinetics model:

$$\chi_{\mathrm{hexane}}=\exp(-k_{\mathrm{dec}}t)$$ 8

$$k_{\mathrm{dec}}=A_{\mathrm{dec}}\exp\left(-\frac{E_{a}}{RT}\right)$$ 9

where χ_hexane_ is the mole fraction of *n*-hexane, *k*_dec_ is the first-order rate constant for decomposition in s^–1^, and *A*_dec_ is the Arrhenius preexponential factor in s^–1^. Based on the study by Li and co-workers^46^ for gas phase pyrolysis of *n*-hexane in a quartz plug flow reactor, the values for *E*_a_ and *A*_dec_ were set to 208.5 kJ·mol^–1^ and 7.98 × 10^11^ s^–1^, respectively. As described in Section 2.3.2, the equivalent volume approximation was used to determine *t* = *t*_e_ at each *T* = *T*_e_.

The results from the lumped decomposition model are shown in Figure 8. To compare this model with our experimental data, we assume that χ_hexane_ can be experimentally estimated from the fractional abundance of the molecular ion (*x*_fa,M+_) as follows:

$$\chi_{\mathrm{hexane}}\approx \frac{x_{\mathrm{fa},M+}(T_{F2})}{x_{\mathrm{fa},M+}(400^{{}^{\circ}}C)}$$ 10

where *x*_fa,M+_(*T*_F2_) is the M^+^ fractional abundance at *T*_F2_. In Figure 8, the literature model (red curve) falls slightly below the experimental results (black points).

**Figure 8:**
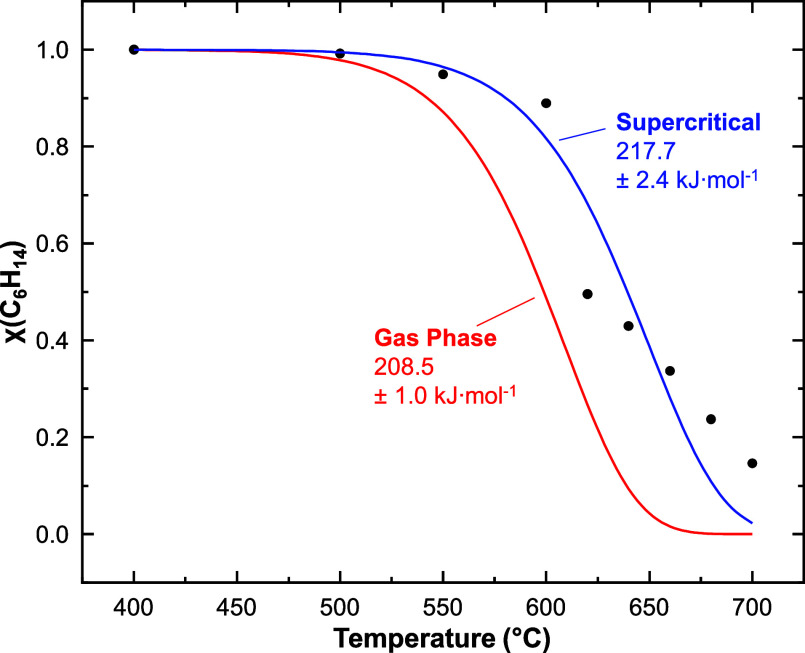
Conversion of *n*-hexane as *T*_F2_ is increased. Experimental mole fractions are plotted as black points. The red curve is based upon a previous gas phase study (*E*_a_ = 208.5 ± 1.0 kJ·mol^–1^).^46^ The blue curve represents the best fit first-order model (*E*_a_ = 217.7 ± 2.4 kJ·mol^–1^).

A nonlinear least-squares fit of the *E*_a_ parameter with *A*_dec_ held constant brought the model into closer agreement with the experimental data (blue curve). Our measured value of *E*_a_ for *n*-hexane decomposition (217.7 ± 2.4 kJ·mol^–1^) is similar to that of Kunzru and co-workers,^7^ which was measured for *n*-heptane at 2.93 MPa (219 kJ·mol^–1^). Similarly, the high-pressure *E*_a_ for *n*-heptane increased from its gas phase value of 208.9 kJ·mol^–1^ at 0.1 MPa.^7^ Note that the errors associated with all least-squares fitting parameters reported herein (e.g., *E*_a_) are standard errors calculated by OriginPro (Version 2023b).

Furthermore, a similar nonlinear least-squares fit of the *n*-hexane consumption data for the 4 mm i.d. reactor (Supporting Information Section 3.3, Figure S9) gave *E*_a_ = 220.7 ± 2.2 kJ·mol^–1^. As the errors of the fitted *E*_a_ values for the 1 and 4 mm reactors overlap, these results further demonstrate both reproducibility and consistency with previous literature. By applying CFD and the equivalent volume approximation, we have characterized the reaction times with sufficient fidelity to estimate the onset temperature of fluid decomposition, which is consistent with the existing literature.^7,46^

### 3.4 Deposition Kinetics

#### 3.4.1 Measurement of Deposition Rates

After successfully characterizing our flow reactor to describe the extent of conversion of the fluid phase reactant, we next focused on correlating the abundance of fluid species detected by online MS to the concomitant deposition rates on the quartz surface. To measure the deposition rates, we applied the Beer–Lambert Law (Section 3.1). Toward this aim, we constructed a calibration plot that relates *A* of the deposit to its average σ, as described in Section 2.2.3. The calibration plot is shown in Figure 9, which displays a strong linear relationship between *A* and σ. The scatter in this plot is most likely random error arising from the uneven distribution of deposit in the tube.

**Figure 9:**
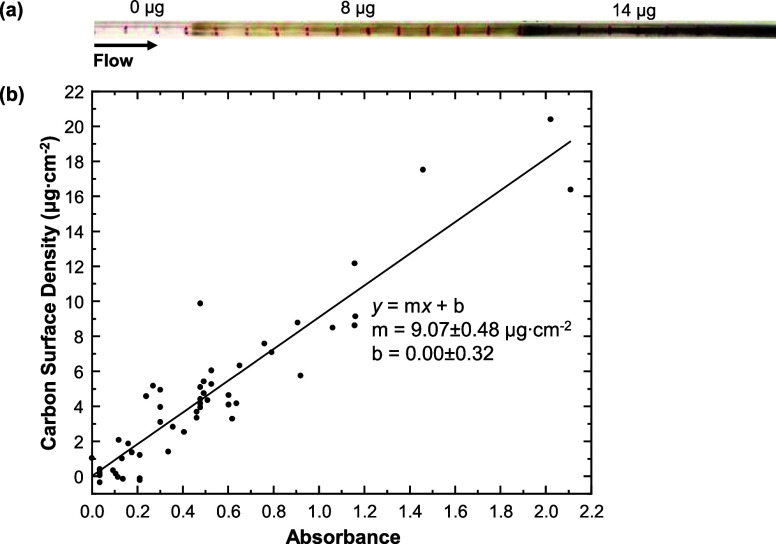
Image of quartz tube (a) and calibration plot (b) to relate optical absorbance to carbon surface density. For reference, the picture in (a) is labeled with the mass of coke in three different sections determined by LECO burnoff. The line of best fit and equation for the calibration curve is shown in (b).

To determine the rate of deposition from the in situ absorbance data in Figure 5, we apply the chain rule:

$$\frac{d\sigma }{dt}=\frac{dA}{dt}\cdot \frac{d\sigma }{dA}=\beta^{-1}\frac{dA}{dt}=m\frac{dA}{dt}$$ 11

where β is defined in ([Disp-formula eq6]) and *m* is the slope of the best fit line from the calibration. Although *A* is influenced by the absorption of both the fluid and the deposit, the contribution from the fluid should be constant if *T*_F2_, *P*, and *t*_e_ are constant. Therefore, $\frac{d\sigma }{dt}$ is directly proportional to $\frac{dA}{dt}$ so that the rate of deposition can be measured at a given *T*.

To examine our experimental data in light of existing deposition models, we obtained absorbance vs. time data for *n*-hexane in separate 1 mm i.d. tubes at four values of *T*_F2_ with *T*_F1_ = *T*_F3_ = 400 °C, *P* = 4.4 MPa, and the inlet flow rate = 0.2 mL·min^–1^ (Figure 10). In these experiments, we began each run with a new tube so that the deposit consistently grew from an inert quartz surface to better compare the deposition rates at different temperatures. At the start of each experiment the flow rate was dropped from 2 to 0.2 mL·min^–1^ to initiate the deposition reaction. In each run, we observed that *T*_F2_ equilibrated as the PID controller adjusted to the slower flow rate within the first 2 min, a time scale much shorter than the experiments. Note that for deposition measurements, the total experiment time is more relevant to the kinetics than the residence time of the fluid in the reactor, *t*_e_, because the deposit is immobile in F2 once it is formed and it grows throughout the experiment.

**Figure 10:**
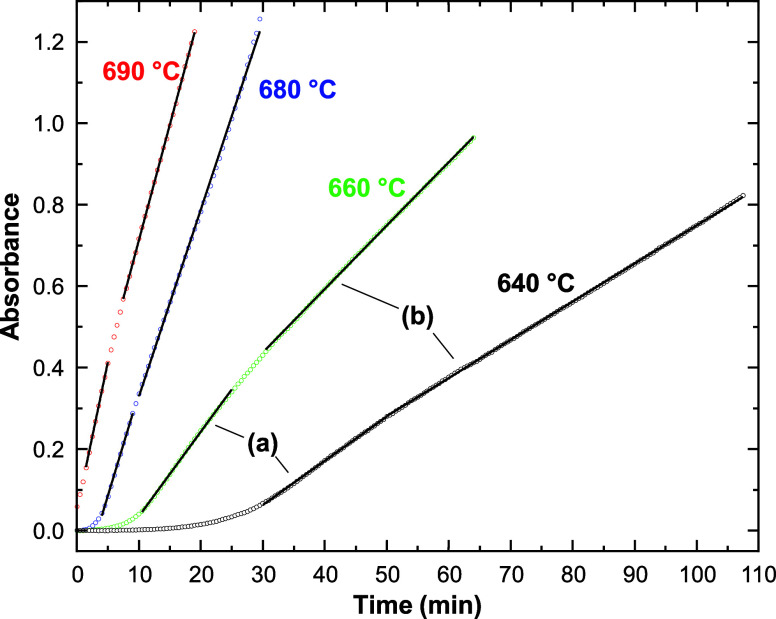
Change in absorbance with increasing experiment time for *T*_F2_ = 640 °C (black), 660 °C (green), 680 °C (blue), and 690 °C (red). Open circles are used for data points, while solid black lines represent linear best fit equations for the regions labeled (a) and (b).

Interestingly, the real-time absorbance measurement reveals that $\frac{d\sigma }{dt}$ is not always constant throughout the experiment. For example, at 640 and 660 °C, sparse deposition is observed until about 30 and 10 min into each run, respectively. It is possible that a longer time is required to form the first monolayer of coke at lower *T* before deposit growth can accelerate. Furthermore, an inflection in the absorbance vs. time data is clearly visible, which is more pronounced at lower *T*. At each *T*, we performed a linear fit of data on both sides of the inflection, labeled (a) and (b) in Figure 10. The values of $\frac{d\sigma }{dt}$ derived from the best fit equations are listed in Table 2. As shown in the Supporting Information (Section 4, Figure S10), the selection of regions (a) vs. (b) had a negligible effect on the activation energies obtained by Arrhenius plots. Therefore, we continue our analysis using the data from region (a).

**Table 2:** Deposition Rates at Different Temperatures for Regions (a) and (b)^a^

*T* (°C)	$\frac{d\sigma }{dt}$ (×10^–1^ μg·cm^–2^·min^–1^)	
	(a)	(b)
640	0.982 ± 0.055	0.850 ± 0.046
660	1.88 ± 0.11	1.412 ± 0.077
680	4.55 ± 0.30	4.17 ± 0.25
690	6.63 ± 0.41	5.15 ± 0.29

a Deposition rates determined from linear best fit equations of data in Figure 10. Standard errors for fitting parameters were calculated using OriginPro (version 2023b).

#### 3.4.2 Evaluation of Purported Deposition Models

With knowledge of deposition rates, we tested various literature models^34,37^ that link coking to the concentrations of fluid phase products [Prod]. These empirical models often have the form

$$n-C_{6}H_{14}\to \mathrm{Prod}\;(e.g.,C_{2}H_{4},C_{3}H_{6},\;\mathrm{and}\;\mathrm{aromatics})$$ 12

Prod→Deposit 13

where it may require multiple steps to link *n*-C_6_H_14_ to Prod depending on the complexity of the mechanism. As such, $\frac{d\sigma }{dt}$ is related to the concentration of one or more cracking products, [Prod], by the following rate equation:

$$\frac{d\sigma }{dt}=A_{\mathrm{dep}}\exp\left(-\frac{E_{a,\mathrm{dep}}}{RT}\right)[\mathrm{Prod}{]}^{n}$$ 14

where *A*_dep_ and *E*_a,dep_ are the Arrhenius parameters for deposition and *n* is the reaction order. To account for [Prod] using the online MS data, we assume that

$$[\mathrm{Prod}]=[R{]}_{0}\chi_{\mathrm{Prod}}\approx \zeta [R{]}_{0}x_{\mathrm{fa},\mathrm{Prod}}$$ 15

where [*R*]_0_ is the initial concentration of reactants, χ_Prod_ is the mole fraction of the product, *x*_fa,Prod_ is the fractional abundance of the product, and ζ is a proportionality constant to estimate the mole fraction of Prod based on its measured fractional abundance by MS. The approximation in ([Disp-formula eq15]) is most reasonable at low conversion levels or if the EI cross sections of the reactant and products are similar.

To determine the experimental *x*_fa*,*Prod_ values for each species at a given *T*, we averaged the raw fractional abundance (*x*_fa,Prod_^raw^) for each species Prod over times in the experiment during which *x*_fa,Prod_^raw^ remained relatively constant and subtracted the estimated EI fragmentation background. Specifically, for the data in Figure 5, *x*_fa,Prod_^raw^ was averaged for at least 5 min near the end of each segment of the *T* ramp.

The approximate fragment background was subtracted as follows:

$$x_{\mathrm{fa},\mathrm{Prod}}(T_{F2})=x_{\mathrm{fa},\mathrm{Prod}}^{\mathrm{raw}}(T_{F2})-x_{\mathrm{fa},M+}^{\mathrm{raw}}(T_{F2})\left[\frac{x_{\mathrm{fa},\mathrm{Prod}}^{\mathrm{raw}}({400\;}^{{}^{\circ}}C)}{x_{\mathrm{fa},M+}^{\mathrm{raw}}({400\;}^{{}^{\circ}}C)}\right]$$ 16

where *x*_fa,M+_^raw^(*T*_*F*2_) is the raw fractional abundance of the *n*-hexane M^+^ ion and *x*_fa,Prod_(*T*_F2_), *x*_fa,Prod_^raw^(*T*_F2_), and *x*_fa,M+_^raw^(*T*_F2_) are written as functions of *T*_F2_. Note that *x*_fa,Prod_^raw^ is defined as the ratio of the integral of a peak (*m*/*z* ±0.5) for species Prod and the integral of the entire mass spectrum. Subtraction of the estimated fragment background was especially important in the case of C_3_H_6_^+^, which can be either the M^+^ ion of propylene or an isobaric EI fragment of C_6_H_14_^+^ at *m*/*z* = 42. Although *x*_fa,C3H6+_^raw^ decreases with *T*_F2_ as *n*-hexane is consumed and the EI fragment is depleted, *x*_fa,C_3_H_6_^+^_ increases with *T*_F2_ because propylene is generated as a product in the fluid. The sum of the fractional abundances (*x*_fa,Prod_^total^) of all products is defined as follows:

$$x_{\mathrm{fa},\mathrm{Prod}}^{\mathrm{total}}=\sum_{\mathrm{Prod}}x_{\mathrm{fa},\mathrm{Prod}}=1-\frac{x_{\mathrm{fa},M+}^{\mathrm{raw}}(T)}{x_{\mathrm{fa},M+}^{\mathrm{raw}}({400\;}^{{}^{\circ}}C)}$$ 17

To apply models to online MS data, it is also assumed that [Prod] does not significantly change between the optical crossing, where the deposition rate is measured, and the mass spectrometer. To validate this assumption, we integrated the equivalent volume ([Disp-formula eq4]) at the optical crossing (2.54 cm below the F2 exit) and calculated χ_hexane_ as a function of *T* at the corresponding *t*_e_ ([Disp-formula eq5]) using the lumped decomposition model from Section 3.3.2. The consumption curve for χ_hexane_ shifted to higher *T* by about 5 °C (Supporting Information, Section 5, Figure S11), which is within the margin of error of the measurement. Therefore, χ at the mass spectrometer is a reasonable estimate for χ at the location of the deposition measurement, greatly simplifying the Arrhenius analysis by allowing us to use *x*_fa,Prod_ from the MS data in ([Disp-formula eq15]) to estimate [Prod].

Substituting ([Disp-formula eq15]) into ([Disp-formula eq14]) we have a revised rate equation:

$$\frac{d\sigma }{dt}=B_{\mathrm{dep}}\exp\left(-\frac{E_{a,\mathrm{dep}}}{RT}\right)x_{\mathrm{fa},\mathrm{Prod}}^{n}$$ 18

$$B_{\mathrm{dep}}=\zeta^{n}[R{]}_{0}^{n}A_{\mathrm{dep}}$$ 19

We treat *B*_dep_ as an adjustable fitting parameter in this work because we do not know the values of ζ needed to convert the literature *A*_dep_ values to *B*_dep_ according to ([Disp-formula eq19]). To measure ζ, calibration plots would be required that relate *x*_fa,Prod_ to [Prod] for each species. However, it is possible to evaluate purported values for *E*_a_ and *n* for a proposed identity of Prod.

Example parameters associated with previous work are listed in Table 3. We also list the *B*_dep_ values derived from nonlinear least-squares fitting of these models to our experimental deposition rate data. A comparison of these models to our experimental data is shown in Figure 11a.

**Table 3:** Parameters for Literature *n*-Hexane Deposition Models and Fitted *B*_dep_ Values

**model** ^a^	**Prod**	***n***	***E***_a**,dep**_ **(kJ·mol** ^**–1**^ **)**	**fitted *B***_**dep**_ ^b^ ^,^ ^c^
Kunzru-I	ethylene	1	127 ± 25	(4.19 ± 0.07) × 10^7^
Kunzru-VII	ethylene	2.4 ± 0.24	98.3 ± 15	(2.83 ± 0.17) × 10^7^
MC-II lateral	aromatics^d^	0.14	80.5	(2.25 ± 0.23) × 10^4^
MC-II catalytic	propylene	1.55	232.3	(1.69 ± 0.05) × 10^14^

a Kunzru-I and -VII models are from ref 34. The MC-II model is from ref 37.

b Standard errors for fitting parameters were calculated using OriginPro (version 2023b).

c The units of *B*_dep_ are μg·cm^–2^·min^–1^·fau^–*n*^, where fau denotes fractional abundance units from the MS measurement.

d Total aromatic content is defined as the sum of benzene, toluene, and xylene/ethylbenzene fractional abundances.

**Figure 11:**
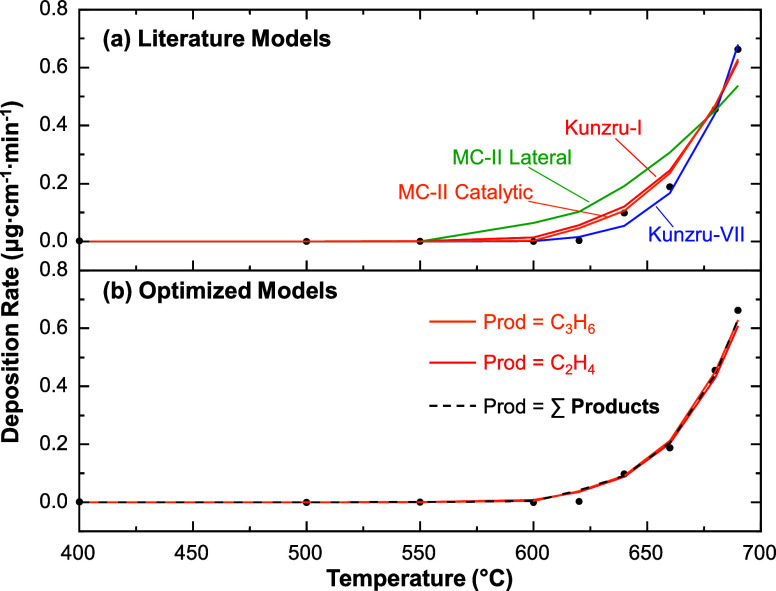
A comparison between models and deposition rate data (black points). Examples of literature models are given in (a), which include Kunzru-I (red),^34^ Kunzru-VII (blue),^34^ MC-II lateral (green),^37^ and MC-II catalytic (orange),^37^ as defined in the manuscript. Optimized models are shown in (b), which were generated by an Arrhenius analysis using Prod = C_2_H_4_ (red), C_3_H_6_ (orange), and ∑Products (dashed) with *n* = 1 and solving for both *E*_a_ and ln­(*B*_dep_).

Based on Figure 11a, several of the literature-based models^34,37^ qualitatively describe the experimental deposition rate data. Both the Kunzru-I and -VII models^34^ and MC-II catalytic model^37^ correctly capture the order-of-magnitude of the deposition rates above *T*_F2_ = 640 °C and reasonably estimate the onset of deposition to within 20 °C. The Kunzru model was developed for gas phase pyrolysis in a high humidity batch reactor, while the MC-II catalytic model was derived to explicitly treat catalytic deposition on a metal surface. Although the conditions in the current work (high pressure and inert quartz reactor) significantly differ, it is noteworthy that these literature models provide useful insight prior to optimization of *E*_a_ or *n*.

In contrast, the MC-II lateral coking model^37^ provides the least adequate description of the data at the current experimental conditions. We chose to separate the lateral and catalytic contributions to the mechanism proposed by Liu and co-workers^37^ because our reactor does not contain a catalytically active surface but may still allow for lateral coking, in which the deposit grows from a preexisting deposit. However, when the MC-II lateral coking model is applied without the catalytic component, the onset of deposition is about 75 °C too low and the curvature is too small to describe the increase in deposition rate with T. This result is a consequence of both the lower *E*_a_ and the smaller reaction order (0.14), which leads to a higher rate at lower *T* despite vanishing levels of aromatic products. Based on this result, it is likely that application of a singular lateral coking mechanism does not effectively describe the initial deposition of amorphous carbon onto the quartz, which dominates the kinetics at lower *T*.

Finally, we tuned the kinetics parameters to optimize the fit for selected Prod species. Toward this end, we generated Arrhenius plots to fit *E*_a_ and ln­(*B*_dep_), which are given in the Supporting Information (Section 6). The results using ethylene and propylene as the product species are plotted in Figure 11b, with the corresponding *E*_a_ and ln­(*B*_dep_) values given in Table 4. To simplify the problem, we chose to focus on first order reactions (*n* = 1) because of the reasonable description of our data using the Kunzru-I model.^34^ Arrhenius plots constructed by assuming numerous reaction orders (*n* = 0, 0.5, 1, 1.5, 2, and 2.5) are shown in the Supporting Information (Section 6, Figure S12).

**Table 4:** Tentative Arrhenius Parameters^a^ for Selected Products with *n* = 1

**product**	***E***_**a,dep**_ **(kJ·mol** ^**–1**^ **)**	**ln(*B***_**dep**_ **)**
ethylene	169 ± 19	22.8 ± 2.4
propylene	263 ± 20	35.1 ± 2.5
∑products	231 ± 16	28.6 ± 2.1

a Standard errors for fitting parameters were calculated using OriginPro (version 2023b).

With the exception of Prod = aromatics, there are a variety of fits that effectively describe the deposition data, and no particular cracking product stands out over the others. In fact, in Figure 11b we illustrate that the data set can be readily fit using *x*_fa,Prod_^total^, an approach that is agnostic to the identity of Prod. As such, our current method is effective for assessing whether a particular deposition model is qualitatively reasonable but cannot be used to derive a single model. In future work, we will calibrate our *x*_fa,Prod_ values to give χ_Prod_ by using off-line gas chromatography with flame ionization detection (GC-FID). This approach will allow us to measure the *A*_dep_ parameters and to refine *E*_a_ values, which will facilitate further down selection between purported models.

## 4 Discussion

Based on the results above, we successfully combined in situ optical spectroscopy in the GTR with online MS to provide real time analysis of the deposition rates and fluid speciation during supercritical *n*-hexane pyrolysis. We have shown that the GTR can be effectively modeled as a simple plug flow reactor by using the equivalent volume method with knowledge of the T and ρ gradients of the fluid along the length of the reactor from 2D-CFD. After applying this approach to estimate *t*_e_, we successfully fitted the conversion of *n*-hexane as a function of *T* to obtain *E*_a_ = 217.7 ± 2.4 kJ·mol^–1^ for the overall fluid phase decomposition, consistent with previous studies.^7,46^ Beyond modeling the fluid phase chemistry, we demonstrated that our deposition data is consistent with several empirical models that relate the fluid speciation to $\frac{d\sigma }{dt}$ .

For the deposition kinetics, we showed that either *x*_fa,ethylene_ or *x*_fa,propylene_ can be used to fit the experimental data, as in the Kunzru^34^ or MC-II^37^ catalytic models, respectively. The *E*_a_ and *B*_dep_ values can be adjusted to bring the empirical fits into yet closer agreement with the deposition rate data by performing Arrhenius analyses for Prod = ethylene or propylene. In fact, we showed that the experimental results can be fitted well by assuming that Prod = ∑products rather than choosing a particular species for Prod. In contrast, we demonstrated that the selection of Prod = aromatics, as in the MC-II lateral coking mechanism, does not adequately describe the measured deposition rates. This result is consistent with the experimental observation that aromatic species emerge at higher temperatures than alkyl cracking products. As evident in Figure 5, benzene, toluene, and xylene appear after deposition is already underway. Therefore, these species are not useful precursors to model the initial stages of deposition at our conditions.

Having established rudimentary models for both the fluid phase cracking of *n*-hexane and coke deposition, the four observable regimes in the *T* ramp experiment (Section 3.2) are easily explained. Regime (2) exists when the rate of fluid phase cracking greatly exceeds the deposition rate because there is a sizable barrier between the cracking products and coke deposit. At higher T, the deposition kinetics accelerate exponentially leading to regimes (3) and (4). As our crude deposition model only includes a single coke precursor, one may expect reaction rates to further increase as polycyclic aromatic hydrocarbons (PAHs) are synthesized and new pathways become accessible to form coke.

In future work, we anticipate more sophisticated models to be applied to the decomposition of hydrocarbon fuel surrogates, which (1) capture supercritical fluid dynamics, (2) predict fluid speciation, and (3) explicitly treat multiple pathways between fluid products and coke. For example, the fluid speciation may be solved using the Reaction Mechanism Generator (RMG) software developed by the Green and West groups.^40,48,49^ Concentrations of the relevant precursors may be input into a secondary model to predict deposition rates at relevant conditions. In this initial study we demonstrate the utility of combining in situ absorption spectroscopy in the GTR with an online MS to rapidly and simultaneously measure both the deposition rate and fluid speciation. By further developing the above methodology, we hope to efficiently identify the onsets of destructive coking for fluid phase hydrocarbons by deriving the **simplest possible models** that capture relationships between initial decomposition and deposition. This work is necessary before more advanced issues such as surface interactions, nonuniform fluid dynamics, and complex mixtures are considered.

## 5 Conclusion

The results above demonstrate that the glass tube reactor (GTR) is an effective tool for unraveling the dynamics of supercritical hydrocarbon decomposition. Using in situ optical spectroscopy, we have correlated the absorbance of the deposit at 405 nm to the surface density of coke on the quartz (μg·cm^–2^·min^–1^). By simultaneously measuring the speciation of the fluid by online MS, we have identified regimes in which cracking occurs with minimal, steady, and rapid deposition. We readily modeled the fluid phase decomposition of *n*-hexane as a lumped first-order process (*E*_a_ = 217.7 ± 2.4 kJ·mol^–1^). For this purpose, we defined *t* by making the equivalent volume approximation^45^ using the values of *T* and ρ_*z*_ computed by 2D-CFD. Finally, we applied several literature deposition models to our data and demonstrated the ability to evaluate and refine such models at our conditions. In future work, we will apply the GTR to mechanistic studies of hydrocarbon degradation, which will be relevant to thermal management applications for the Air Force.

## Supplementary Material



## Notes

Supplemental optical absorption spectra; effect of heated transfer lines on product distribution; results from the 4 mm i.d. reactor; effect of changes in deposition rates on Arrhenius plots; validation of MS product abundance measurement near the optical crossing; and Arrhenius plots for different products and reaction orders (PDF)

## Notes

DISTRIBUTION STATEMENT A. Approved for public release. Distribution is unlimited. AFRL-2025-5759.
