# Supplementary material for: Online Chemical Analysis of Flowing n‑Hexane in a Pyrolysis Reactor by Optical Spectroscopy and Molecular Beam Mass Spectrometry
Source: J Phys Chem A. 2026 Mar 9;130(11):2460–72. doi: 10.1021/acs.jpca.6c00148 (PMC13007033; doi:10.1021/acs.jpca.6c00148)
Supplement: Supplementary file 1 [file jp6c00148_si_001.pdf]

Supporting Information

Online Chemical Analysis of Flowing *n*-Hexane in a Pyrolysis Reactor by Optical Spectroscopy and Molecular Beam Mass Spectrometry

Matthew C. Rohan,[a,b] Cole J. VanDyke,[a,b] Michael S. Hanchak,[a,b] Elizabeth M. Craft,[a,b] Elizabeth S. Kurian,[a] Alexander D. Tucker,[a] William K. Lewis,[a*] and Andrew F. DeBlase[a,b*]
[a] Air Force Research Laboratory, Aerospace Systems Directorate, Wright-Patterson Air Force Base, Dayton, OH 45433
[b] University of Dayton Research Institute, Dayton, OH 45469

Table of Contents

1 Supplemental Optical Absorption Spectra 1
2 Effect of Heated Transfer Lines on Product Distribution 3
2.1 Stainless Steel Tube Rig Apparatus 3
2.2 External vs. Internal Pyrolysis Reactor 3
3 Results from the 4 mm i.d. Reactor 5
3.1 Experimental Results 5
3.2 Reproducibility Study 7
3.3 CFD Results 7
4 Effect of Changes in Deposition Rates on Arrhenius Plots 10
5 Validation of MS Product Abundance Measurement Near the Optical Crossing 11
6 Arrhenius Plots for Different Products and Reaction Orders 12
References 13

1 Supplemental Optical Absorption Spectra

In Figure S1, optical spectra are presented for fluid samples extracted from the GTR. As high as 690 °C, the color change is difficult to see by eye. However, a weak absorbance occurs below 450 nm for T_{F2} > 680 °C. Note that the decreasing volume of liquid as T increases results from the high liquid to gas conversion at these temperatures. The absorbance for the sample with T_{F2} = 680 °C increases as λ decreases from 450 to 400 nm, which is a similar behavior as the spectrum of the sample from the waste container.

* Corresponding author emails: william.lewis.49@us.af.mil and andrew.deblase.ctr@us.af.mil
DISTRIBUTION STATEMENT A. Approved for public release. Distribution is unlimited. AFRL-2025-5759

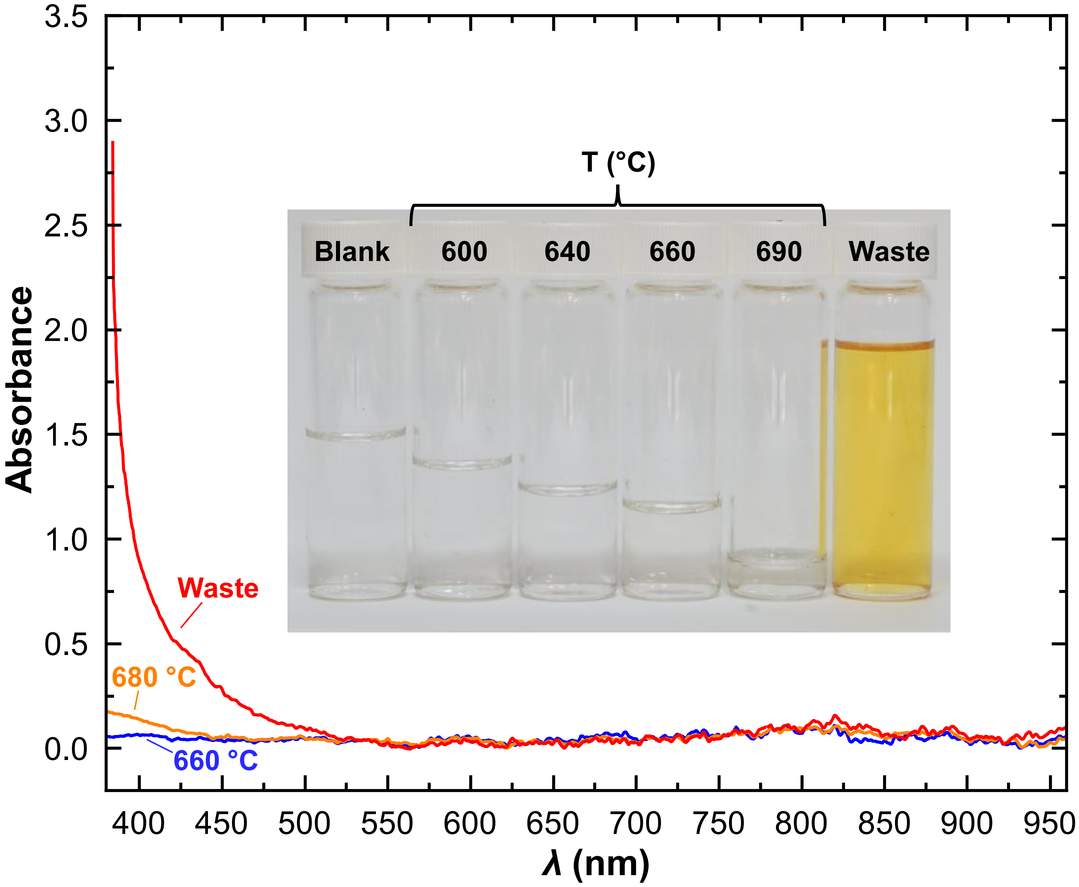

Figure S1. Absorbance spectra of fluid samples obtained at the exit of the GTR during an experiment with $T_{F1} = T_{F3} = 400$ °C and T_{F2} ramped. Spectra are shown for $T_{F2} = 660$ °C, $T_{F2} = 680$ °C, and for the cumulative waste. The waste was collected over multiple runs and was more concentrated in the chromophore because the fluid was allowed to evaporate from the collection bottle.

DISTRIBUTION STATEMENT A. Approved for public release. Distribution is unlimited. AFRL-2025-5759

2 Effect of Heated Transfer Lines on Product Distribution

2.1 Stainless Steel Tube Rig Apparatus

To test the necessity of performing pyrolysis in the supersonic expansion nozzle, a stainless-steel tube rig (STR) was constructed. This rig contained a pyrolysis furnace external to the mass spectrometer, which consisted of a 26.7 cm section of 3.2 mm o.d. stainless steel tubing that was tightly wound with a 61 cm long piece of heat tape. The external furnace reached temperatures as high as 760 °C. The fuel surrogate (*n*-dodecane) flowed through this heated section to initiate pyrolysis and was subsequently cooled to about 250 °C in heated transfer lines that connected the external furnace to the mass spectrometer. The time in the heated transfer lines was on the order of several minutes. Two of these heated transfer lines were made from 1.6 mm o.d. stainless steel tubing wrapped in 122 cm long pieces of heat tape. The test fluid was *n*-dodecane (Thermo Fisher, >99%), which was filtered (0.5 µm inline Swagelok filter) before it entered the supersonic expansion nozzle to remove coke particles that could plug the 10 µm nozzle. Prior to the experiment, the fluid was purged with N_2 to eliminate the effects of autoxidative coking. The temperatures of the external furnace and heated transfer lines were controlled using proportional-integral-device (PID) temperature controllers (BriskHeat). In this experiment, we used the 3.2 mm o.d. supersonic expansion nozzle design with a 10 µm orifice, described in our previous work.[1-4] We ran the internal furnace at 420 °C to assure single-phase flow into the instrument at a temperature below the pyrolysis threshold. The fluid was pushed through the rig using N_2 buffer gas at 600 psig and its flow rate was measured to be about 0.1 mL·min^{-1} using a Hastings flow meter (300 Vue). A diagram and picture of the STR is given in Figure S2.

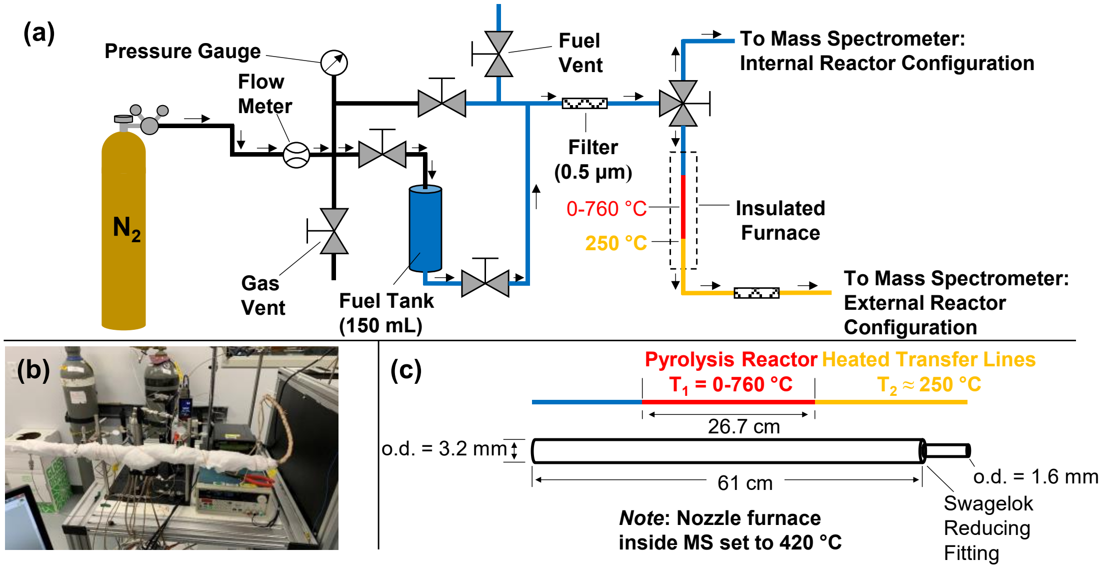

Figure S2. The STR apparatus. A schematic showing the path of the fuel through the system is given in (a). A photograph of the external furnace reactor is depicted in (b), while dimensions and temperatures of the reactor are illustrated in (c).

2.2 External vs. Internal Pyrolysis Reactor

Our previous method[1-4] allowed us to probe chemical species in a pyrolytic reaction mixture *in situ* because the fuel/fuel surrogate was directly sampled at extreme temperature and pressure.

Previously, we observed that a variety of saturated hydrocarbon fuel surrogates undergo pyrolysis to a similar aromatic product distribution[2] at the most extreme temperatures prior to obstruction of the supersonic expansion nozzle. These results are reproduced for convenience in Figure S3.

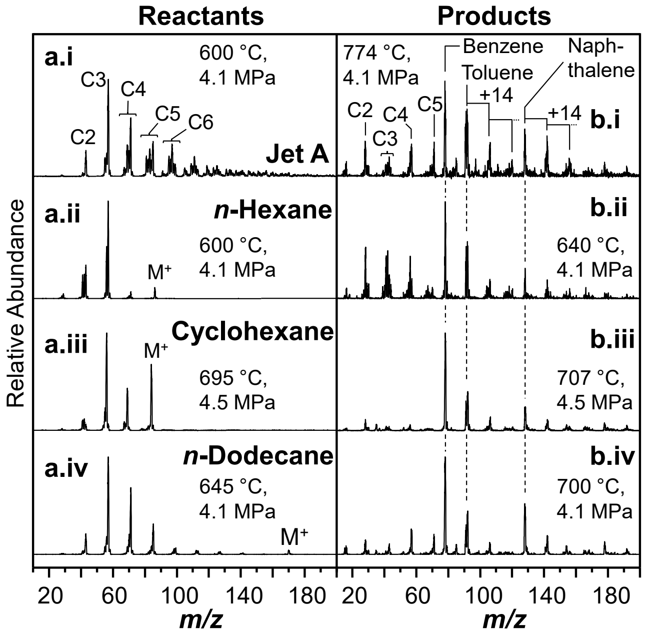

Figure S3. Mass spectra of reactant fuel/surrogates (a) and final products at extreme T and P (b). The different fuels/surrogates studied included Jet A (i), n-hexane (ii), cyclohexane (iii), and n-dodecane (iv). Major species are labeled, and T and P conditions are shown above each plot.

Despite the ability of the previous apparatus to sample *in situ* product distributions, plugging of the supersonic expansion nozzle was a significant technical challenge, which resulted in a loss of control over the flow rate of the fluid. Furthermore, optical access would be significantly more challenging in the previous design because deposition occurred inside the stainless-steel nozzle. Therefore, we evaluated the necessity of maintaining the fuel at pyrolytic temperatures as it is passed through the supersonic expansion nozzle.

Figure S4 shows results of a study in which *n*-dodecane at 600 psig was heated to 760 °C (measured on the outer surface of the stainless steel tube) using the external furnace of the STR. The product distribution obtained at these conditions (Figure S4a) is very similar to that observed in our previous work[2] (Figure S4b) at conditions in which nearly complete conversion of the *n*-dodecane reactant was achieved. Also, the aromatic product distribution is consistent with the spectra of the other compounds and Jet A, which are shown in Figure S3.

Although small molecular ion peaks of some larger PAHs were observed in our previous study (chrysene, perylene, *etc.*) and not when using the STR, much can still be learned about the initial formation of aromatic compounds (*e.g.*, benzene, toluene, and naphthalene) because the relative abundances of these products do not greatly differ within the experimental error. Regardless, the *n*-hexane study in the GTR describes a much lower degree of conversion (*n*-hexane reactant still detectable at T ≤ 700 °C), implying that these larger PAHs are likely to be insignificant fluid phase products. Clearly, there is a substantial benefit to filtering the fuel prior to the

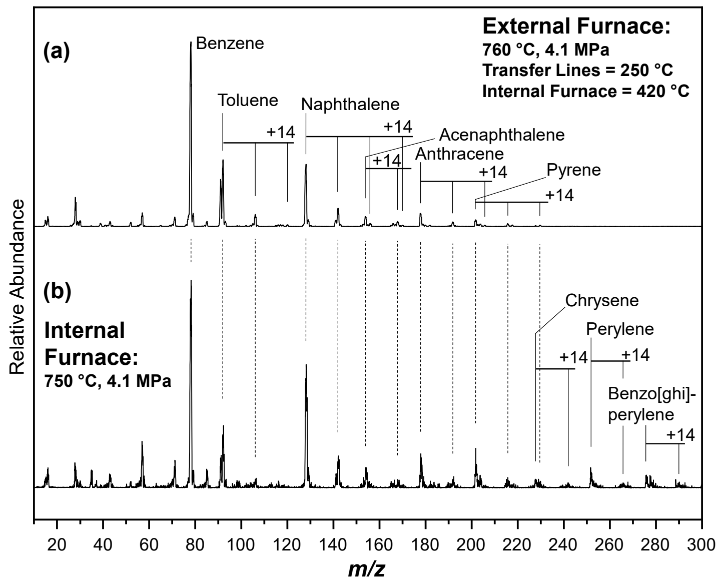

Figure S4. Mass spectra showing final product distributions when *n*-dodecane was pyrolyzed using the external furnace of the STR (a) and internal furnace from the previous work[4] (b). Conditions are given above each spectrum and major species are labeled.

supersonic expansion nozzle to prevent plugging and to maintain a controlled flow rate. This advantage far outweighs the fine speciation details that may be lost for high-order products, which are beyond the scope of the current study. Note that the product distribution was stable for over an hour when running the STR at 760 °C, whereas PAHs could only be observed for a few minutes before the nozzle plugged in the previous work[1-4].

3 Results from the 4 mm i.d. Reactor

3.1 Experimental Results

Results from the T ramp experiment for *n*-hexane in a 4 mm i.d. quartz tube are shown in Figure S5. Apart from the tube i.d. and T_{F2} values, all other conditions matched those reported in Figures 4 and 5 ($T_{F1} = T_{F3} = 400$ °C, P = 4.4 MPa, inlet flow = 0.2 mL·min^{-1}). As expected, the larger reactor volume, which corresponds to a longer reaction *t*, results in a lower onset T for cracking in the 4 mm i.d. reactor when compared to the 1 mm i.d. reactor (≈ 500 vs. 560 °C). Also, the onset of deposition is much lower in the case of the 4 mm i.d. reactor (≈ 580 vs. 640 °C).

Unlike the 1 mm i.d. reactor, in the 4 mm i.d. reactor it takes a longer time for the MS relative abundances to stabilize. In each step, product abundances increase and decrease in intensity before becoming constant, which seems to correspond to the temperature overshoot of the PID controller each time T_{F2} is changed. These overshoot periods are shorter and far less pronounced in the case of the 1 mm i.d. reactor. As the residence time is about an order of magnitude larger in the 4 mm i.d. reactor, the diffusion of products likely lengthens the equilibration time for each step in the ramp.

Although the onset T of deposition decreases for the 4 mm i.d. reactor when compared to the 1 mm i.d. tube, the change in $\frac{d\sigma}{dt}$ for the 4 mm i.d. reactor does not occur immediately as T_{F2} is increased to 580 °C. Interestingly, in Figure 10 we observe a similar delay in deposition for the 1 mm reactor when T_{F2} = 640 and 660 °C for separate runs starting with clean quartz tubes. One possible explanation for both these results is that it takes a longer time for the deposit to seed at the lower temperatures on the noninteracting quartz surface. When the first monolayer forms, the rate may subsequently accelerate. In the case of the 4 mm i.d. tube, fluid phase conversion is much higher at lower T because of the longer reaction *t*. Furthermore, the probability of a nucleation event is likely higher in case of the 4 mm i.d. tube because the surface area is four times greater. If deposit precursors are in higher concentrations, the increase in deposition rate should be more dramatic when nucleation of the deposit occurs. The deposition rate may also depend on concentration gradients of products along the 2 mm reactor radius, which are likely influenced by diffusion. This scenario would explain the more sudden and sporadic onset of rapid deposition in the 4 mm i.d. reactor.

The purpose of this study is to demonstrate a simple, easily characterized, and well controlled plug flow reactor (PFR) to study the dynamics of deposition and its relationship to fluid phase cracking. Thus, we focus on the 1 mm i.d. quartz tube results in the manuscript to avoid explicit treatment of the more complex fluid dynamics at play in the 4 mm i.d. reactor.

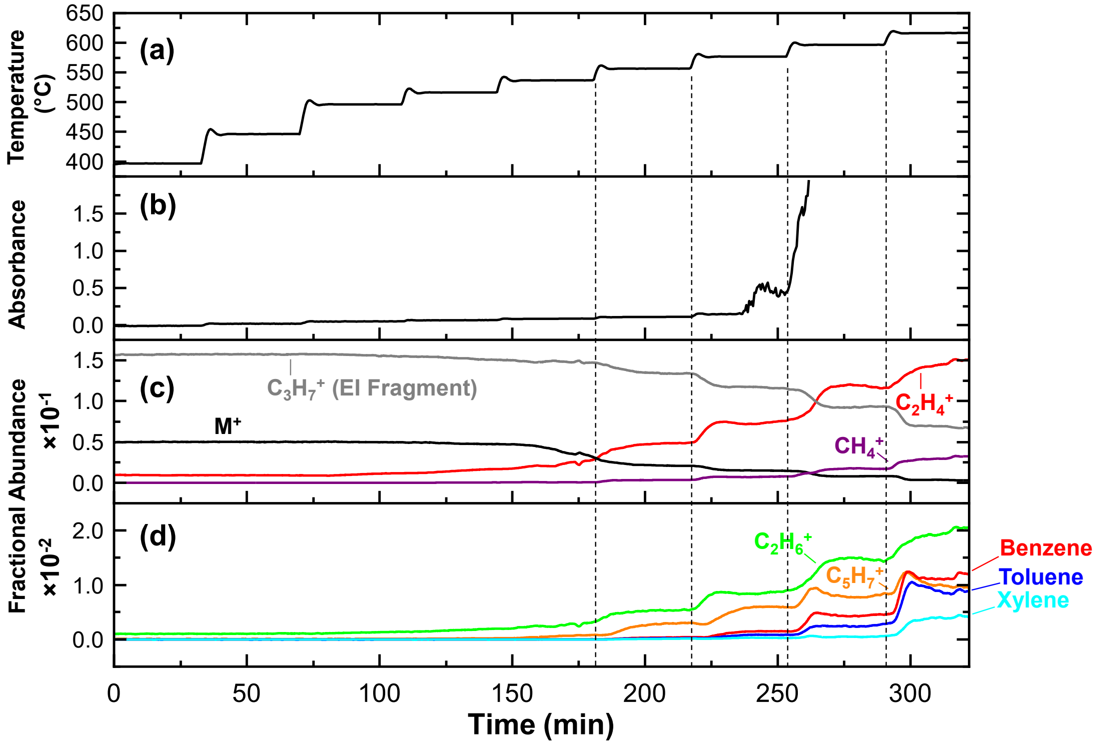

Figure S5. Experimental results from the GTR with a 4 mm i.d. quartz tube reactor at 4.4 MPa and an inlet flow rate of 0.2 mL·min^{-1}. The wall temperature of F2 is plotted in (a) as a function of experiment time. The absorbance of 405 nm light through the quartz tube is given in (b). The fractional abundances of key species measured by MS are color coded and plotted in (c) and (d). The ordinate axis is expanded ×10 in (d) relative to (c) to better visualize minor ions.

Nevertheless, the shifts to lower T for cracking chemistry and deposition in the 4 mm i.d. reactor are instructive when evaluating the simple model presented in the manuscript.

3.2 Reproducibility Study

To demonstrate the reproducibility of our data, we performed duplicate survey scans for *n*-hexane in the 4 mm i.d. reactor with T_{F1} = 400 °C, T_{F2} = 375 – 640 °C, T_{F3} = 375 °C, and an inlet flow rate of 0.5 mL·min^{-1}. The temperature profiles from the two runs are shown in Figure S6a, while the fractional abundances of key ions are plotted in Figures S6b and S6c. Based on comparing the solid and dashed traces, the MS data is reasonably reproducible. The small shift in the fractional abundances with time corresponds to the shift in the temperature profile by several minutes between Runs 1 and 2.

As the MS data is quite stable over time, the largest source of experimental error is likely the reproducibility of conditions between different runs. To minimize this error, data in the manuscript was recorded using a PID temperature controller on F2 (Variac heater used for Figure S6). In the manuscript (Figure 5), smaller temperature steps (20 °C) were made and sufficient time elapsed so that the product distributions were stable at each temperature before the subsequent step. Therefore, we believe that the reproducibility in Figure S6 represents a worst-case scenario, which is reasonable, nevertheless.

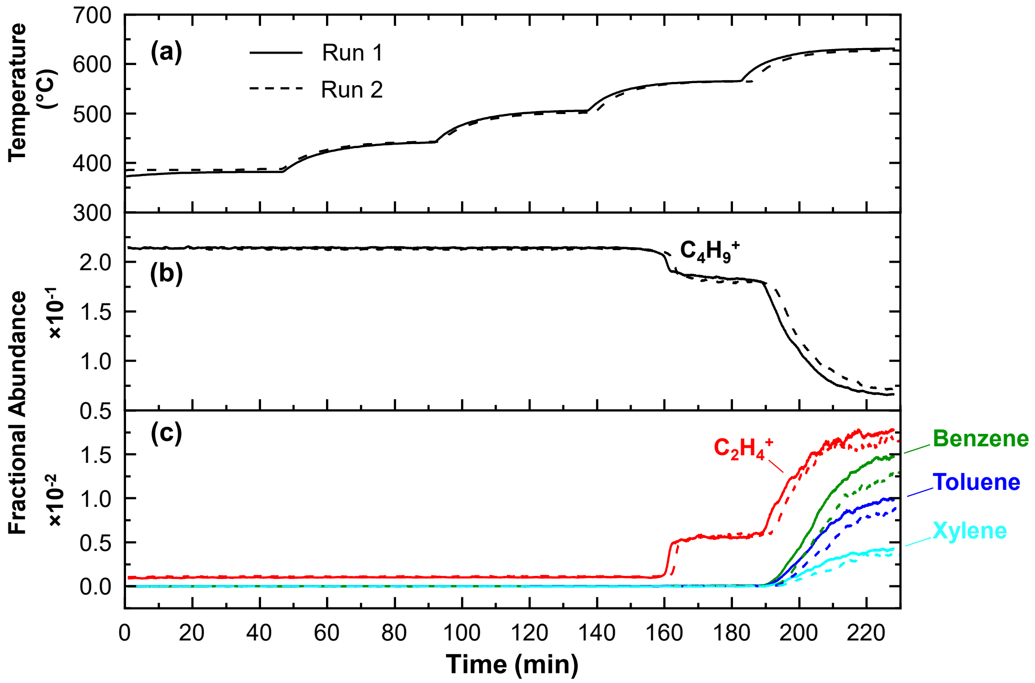

Figure S6. Reproducibility study for MS data from the GTR. The temperature profiles are given in (a), while the fractional abundances of key ions are shown in (b) and (c). The solid traces represent data from Run 1 while the dashed traces correspond to Run 2. Fractional abundances corresponding to different ions are color coded and labeled.

3.3 CFD Results

Figure S7 compares RMG simulations for 1 and 4 mm i.d. quarts tube reactors. In this example, the conditions are the same for both reactors with T_{F1} = T_{F3} = 400 °C and T_{F2} = 600 °C,

P = 4.4 MPa, and an inlet *n*-hexane flow rate of 0.2 mL·min^{-1} at 25 °C. As anticipated, it takes longer for the fluid temperature to equilibrate with the reactor wall for the 1 mm reactor. This result is consistent with the smaller volume, which reduces the residence *t* in the 1 mm tube for the same inlet flow rate as the 4 mm tube. In both cases, the mean radial T, $\langle T_r \rangle$, equilibrates with the wall faster than the centerline T, T_c, does because T_c represents the worst-case scenario in which heat must be transferred throughout the full radius of the reactor.

The inflection in $\langle T_r \rangle$ of the 4 mm tube (blue dots) between F2 and F3 is likely an effect of buoyancy. Note that the GTR is vertically oriented with F3 on top. As T drops between F2 and F3, the hotter and less dense fluid near the wall flows upwards, which warms the fluid downstream in F3 and cools the fluid closer to the outlet of F2. This phenomenon is not observed in the case of the 1 mm reactor because the fluid velocity is much greater. To estimate product distributions, we reduced the dimensionality of the problem by assuming that the fluid T is equal to $\langle T_r \rangle$.

In Figure S8, we summarize the 2D-CFD simulation results by plotting the warm-up time ($t_{\text{warm-up}}$) and time at maximum T (t_{Tmax}) as bar charts for all values of T_{F2} in both the 1 mm (a) and

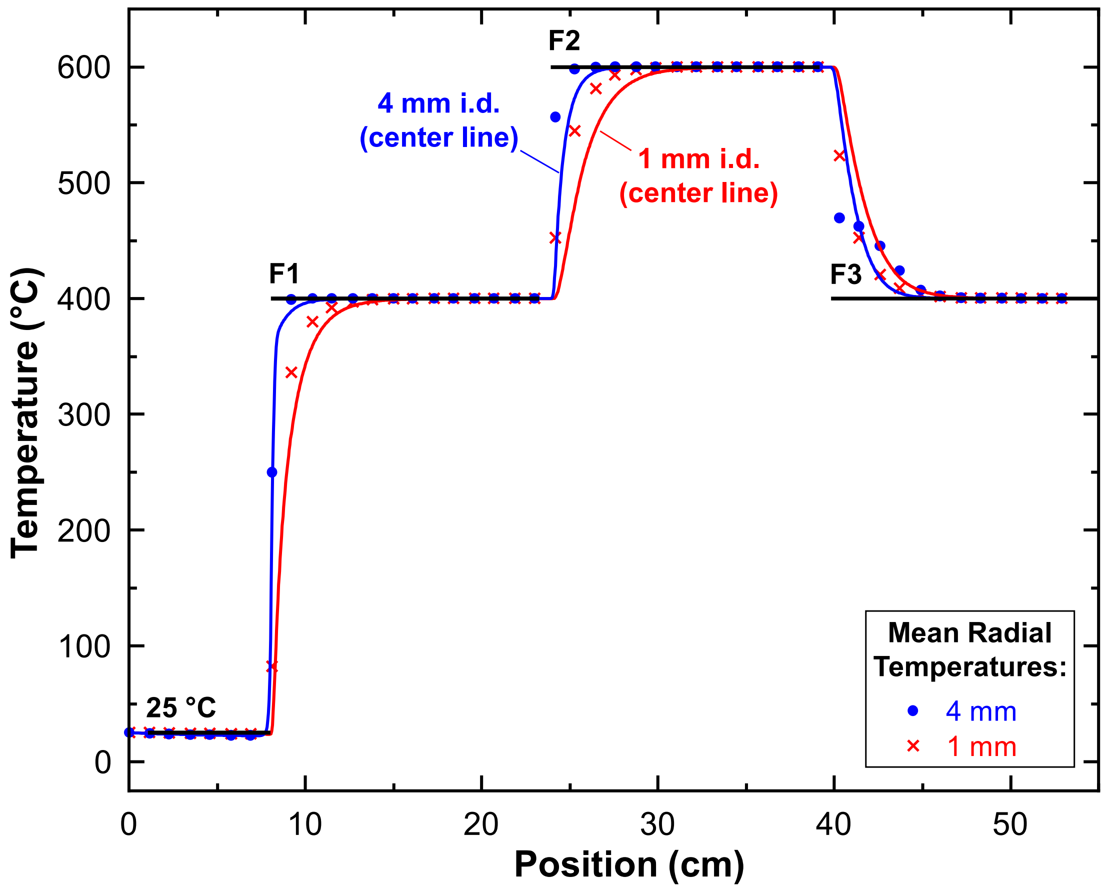

Figure S7. Temperature *vs*. position profile in the GTR calculated by 2D-CFD for 1 mm (red) and 4 mm (blue) i.d. quartz tube reactors. Simulations were performed for *n*-hexane with an inlet flow rate of 0.2 mL·min^{-1} and P = 4.4 MPa. The black lines denote the T setpoints for the inlet fuel and furnaces. The solid curves represent the centerline temperatures. The x's and dots indicate the mean radial temperatures for the 1 and 4 mm i.d. tubes, respectively, that were calculated at discrete positions along the reactor.

DISTRIBUTION STATEMENT A. Approved for public release. Distribution is unlimited. AFRL-2025-5759

4 mm (b) i.d. quartz reactors. In both cases, as T_{F2} increases, $t_{warm\text{-}up}$ and t_{Tmax} decrease such that the fractional contribution of $t_{warm\text{-}up}$ to the total time (t_{F2}) increases. Consistent with the results in Figure S7, $t_{warm\text{-}up}$ contributes much more significantly to t_{F2} for the 1 mm reactor because t_{F2} is an order of magnitude smaller.

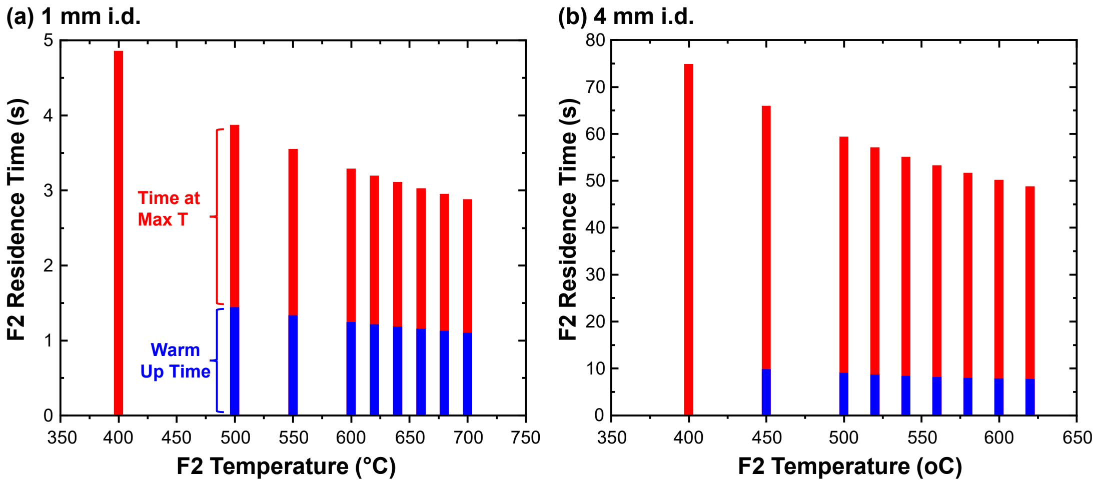

Figure S8. Total residence time in F2 (t_{F2}) and its contributions from $t_{warm\text{-}up}$ (blue) and t_{Tmax} (red) for CFD simulations with different values of T_{F2} in the case of the 1 mm (a) and 4 mm (b) i.d. quartz reactors.

In Table S1, we list values for t_{Tmax}, t_{F2}, and the residence time making the equivalent volume approximation[5] (t_e) for the 4 mm i.d. reactor. As is the case for the 1 mm i.d. reactor, t_e is between t_{Tmax} and t_{F2}. Note that when T_{F2} = 450 °C, $t_e > t_{F2}$; however, this exception is attributed to the similar values of $T_{F1} = T_{F3}$ = 400 °C and T_{F2}. Realistically, the longer t_e at T_{F2} = 450 °C is inconsequential because the overall reaction rate is negligible at this T.

Table S1: Values of t_{F2}, t_{Tmax}, and t_e Derived from 2D-CFD Simulations for *n*-Hexane in a 4 mm I.D. Quartz Tube Reactor

T (°C)	t_{F2} (s)	t_{Tmax} (s)	t_e (s)
400	74.8	74.8	-
450	65.9	56.1	74.3
500	59.3	50.4	56.3
520	57.1	48.4	53.9
540	55.0	46.7	52.0
560	53.2	45.1	50.3
580	51.6	43.7	48.7
600	50.1	42.3	47.3
620	48.7	41.1	45.9

DISTRIBUTION STATEMENT A. Approved for public release. Distribution is unlimited. AFRL-2025-5759

Using T_e and t_e from the equivalent volume approximation, we fitted the fluid phase *n*-hexane decomposition data to determine E_a as described in the manuscript (Section 3.3.2). Results are shown for both the 1 and 4 mm i.d. reactors in Figure S9. The E_a values are 217.7 ± 2.4 and 220.7 ± 2.2 kJ·mol^{-1} for the 1 and 4 mm i.d. reactors. These results are within the margin of error of the two fits, demonstrating a consistent E_a for both quartz reactors. Therefore, the shift in the decomposition curve to lower temperature for the 4 mm reactor relative to the 1 mm reactor is consistent with the longer t_e in the 4 mm reactor.

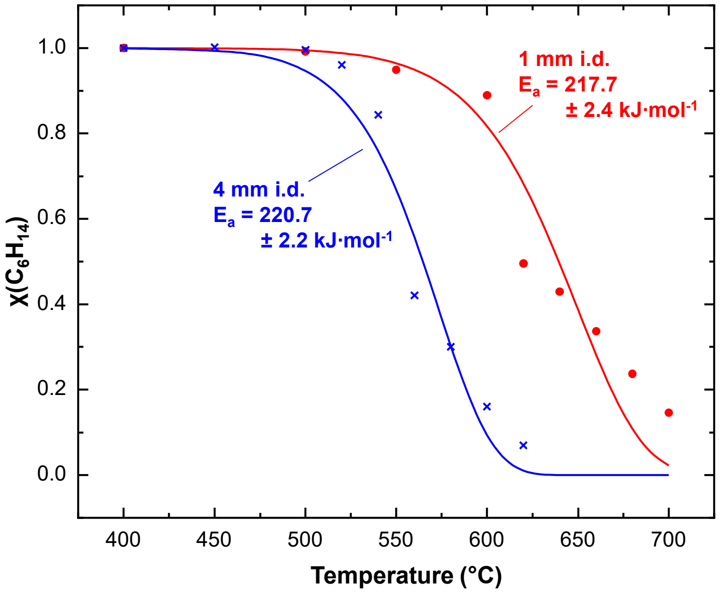

Figure S9. Comparison of the calculated χ for *n*-hexane and the measured fractional abundance of the molecular ion for the cracking of *n*-hexane in 1 mm (red) and 4 mm (blue) i.d. reactors. Experimental measurements are denoted by the points and x's for the 1 and 4 mm i.d. reactors, respectively. The conditions were as follows: P = 4.4 MPa, inlet flow = 0.2 mL·min^{-1}, and F1 = F3 = 400 °C.

4 Effect of Changes in Deposition Rates on Arrhenius Plots

In Figure S10, two Arrhenius plots are made using the data shown in Figure 10 of the manuscript in the regions labeled (a) and (b). In these Arrhenius plots, we assume that $n = 0$. Both plots yield very similar values for E_a and Arrhenius constants. The Arrhenius parameters from regions (a) and (b) fall within the errors of each other. Therefore, we conclude that the visible inflections in the absorbance data in Figure 10 do not significantly change E_a or A_0.

DISTRIBUTION STATEMENT A. Approved for public release. Distribution is unlimited. AFRL-2025-5759

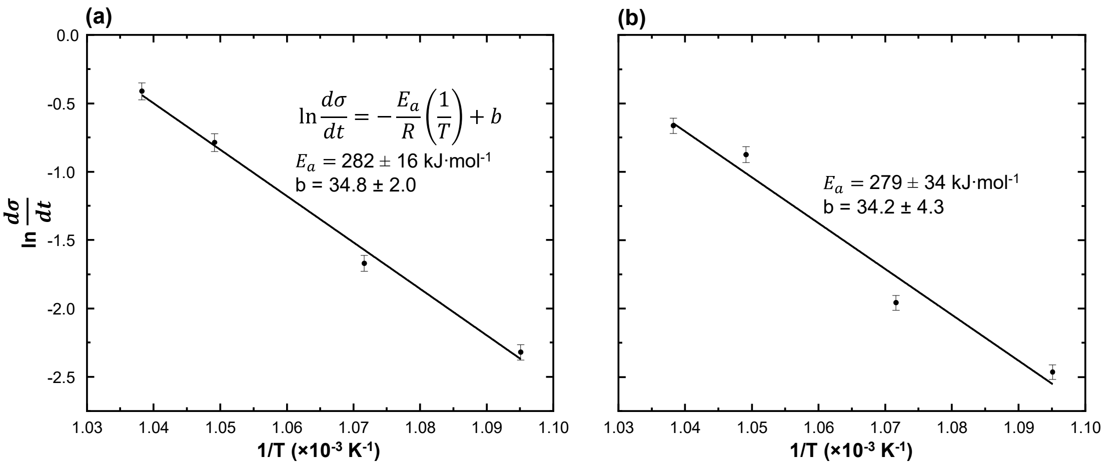

Figure S10. Arrhenius plots constructed from regions (a) and (b) in Figure 10 of the manuscript. The reaction order is assumed to be 0 for this comparison.

5 Validation of MS Product Abundance Measurement Near the Optical Crossing

To validate that the MS measurement adequately represents the product concentration near the optical crossing, where the deposition measurement is made, we applied the equivalent volume approximation to compute t_e at the optical crossing. In Figure S11, the calculated consumption of *n*-hexane is plotted for both t_e at the optical crossing and t_e corresponding to the full reactor. The consumption curve is shifted by about 5 °C to higher T, consistent with the slightly reduced

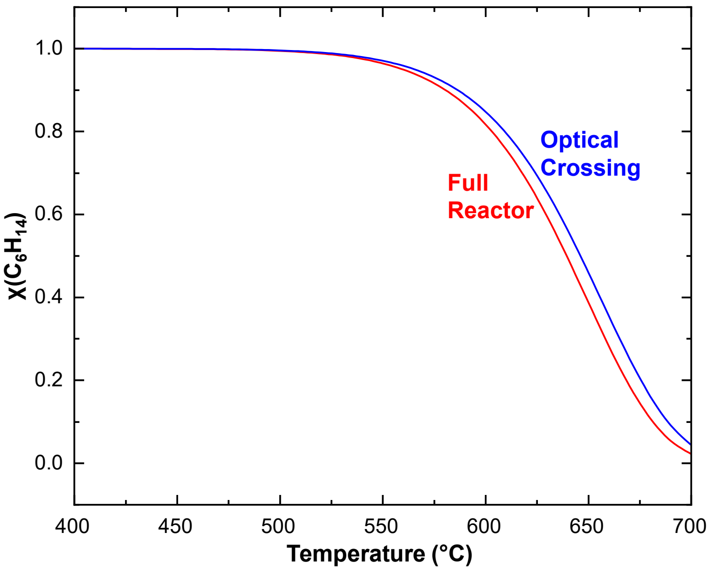

Figure S11. Comparison of reactant consumption plots for V_e and t_e calculated at the optical crossing (blue) and over the full length of the reactor (red). The fluid is n-hexane with P = 4.4 MPa, inlet flow rate = 0.2 mL·min^{-1}, and $T_{F1} = T_{F3}$ = 400 °C.

DISTRIBUTION STATEMENT A. Approved for public release. Distribution is unlimited. AFRL-2025-5759

residence time at the optical crossing (2.54 cm below reactor exit). As this effect is minimal, we assume that the product distributions measured by online MS sufficiently represent the abundances of species at the location where the deposition rate is measured.

6 Arrhenius Plots for Different Products and Reaction Orders

To examine the effect of the identity of Prod and the reaction order on the Arrhenius plot, we tested cases where Prod = ethylene, propylene, aromatics, and ∑products. Because we do not have sufficient data to fit all three parameters (E_a, B_{dep}, and n), we generated separate Arrhenius plots corresponding to n = 0, 0.5, 1, 1.5, 2, and 2.5.

As described in the manuscript, the general rate law for deposition from a species Prod is

$$\frac{d\sigma}{dt} = B_{\text{dep}} \exp\left(-\frac{E_{a,\text{dep}}}{RT}\right) x_{fa,i}^{n}. \quad (1)$$

Dividing both sides by $x_{fa,i}^{n}$ and taking the natural log, we have

$$\ln\left(\frac{1}{x_{fa,\text{Prod}}^{n}} \frac{d\sigma}{dt}\right) = -\frac{E_a}{R} \cdot \frac{1}{T} + \ln(B_0)\,, \quad (2)$$

where a plot of $\ln\left(\frac{1}{x_{fa,\text{Prod}}^{n}} \frac{d\sigma}{dt}\right)$ *vs.* $\frac{1}{T}$ should have a slope of $-\frac{E_a}{R}$ and intercept of $\ln(B_0)$. A series of such Arrhenius plots for different values of n and identities of Prod are given in Figure S12. Arrhenius parameters for the plots with integer reaction orders are tabulated in Table S2.

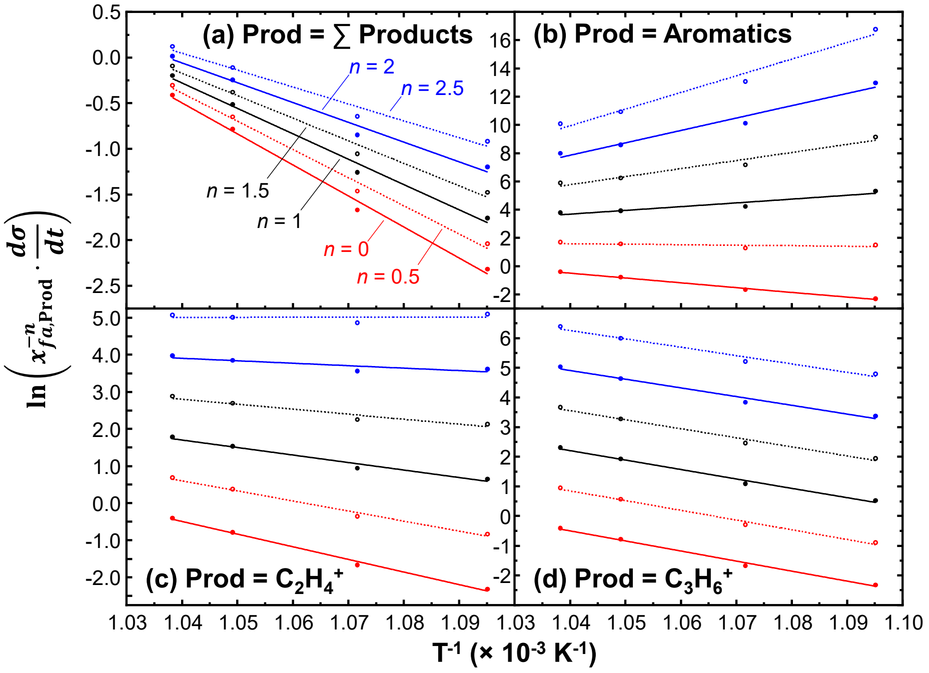

Figure S12. Arrhenius plots generated using different species as the essential intermediate, Prod, preceding deposition: (a) sum of all products, (b) sum of aromatics, (c) ethylene, and (d) propylene. Plots are shown for different orders of reaction: n = 0 (red, solid), 0.5 (red, dotted), 1 (black, solid), 1.5 (black, dotted), 2 (blue, solid), and 2.5 (blue, dotted).

DISTRIBUTION STATEMENT A. Approved for public release. Distribution is unlimited. AFRL-2025-5759

Table S2: Fitted Arrhenius Parameters for *n*-Hexane Deposition in a 1 mm i.d. Quartz Tube Using Different Species, Prod, and Reaction Orders, *n*

	***n* = 0**		***n* = 1**		***n* = 2**	
**P Prod**	**E_a***	**ln(B_0)†**	**E_a***	**ln(B_0) †**	**E_a***	**ln(B_0) †**
∑ products	282 ± 16	34.8 ± 2.0	231 ± 16	28.6 ± 2.1	180 ± 17	22.4 ± 2.2
Aromatics only	282 ± 16	34.8 ± 2.0	-223 ± 51	-24.2 ± 6.6	-728 ± 87	-83 ± 11
$C_2H_4^+$ only	282 ± 16	34.8 ± 2.0	169 ± 19	22.8 ± 2.4	55 ± 23	10.8 ± 2.9
$C_3H_6^+$ only	282 ± 16	34.8 ± 2.0	263 ± 20	35.1 ± 2.5	244 ± 24	35.5 ± 3.1

*E_a has units of kJ·mol^{-1}. † B_0 has units of μg·cm^{-2}·min^{-1}·fau^{-n}, where fau denotes fractional abundance units from the MS measurement.

As discussed in the manuscript (Section 3.4.2), there are a variety of ways to fit the experimental deposition data. Our data confirms that the choice of Prod = ethylene, propylene, or ∑products all have merit. However, Prod = aromatics leads to a negative activation energy except for the cases of n = 0 and 0.5. This result is consistent with the detection of a deposit prior to the appearance of aromatic species in Figure 5 and implies that the detection of aromatic precursors is insufficient for predicting the earliest stages of deposition. However, the presence of aromatics in the mass spectrum serves as a warning that deposition is already under way.

References

1. Bunker, C. E.; DeBlase, A. F.; Youtsler, T. A.; Sanders, N. L.; Lewis, W. K. Enhanced Bimolecular Reaction in a Two-Component Fluid Under Pyrolytic Conditions: In Situ Probing of the Pyrolysis of Jet Fuel Surrogates Using a Supersonic Expansion Molecular Beam Mass Spectrometer. *Energy Fuels* **2018,** *32* (3), 3391-3398.
2. DeBlase, A. F.; Bruening, C. R.; Lewis, W. K.; Bunker, C. E. Probing the Supercritical Pyrolysis Regime by Mass Spectrometry: The Effects of Aliphatic Versus Aromatic Content on the Composition of an Endothermic Fuel. *Energy Fuels* **2018,** *32* (12), 12289-12297.
3. DeBlase, A. F.; Bruening, C. R.; Lewis, W. K.; Bunker, C. E. In Situ Diagnostic of Supercritical Fuel Surrogates: Probing Heterogeneous Catalysis by Collision-Induced Dissociation in a Molecular Beam Tandem Mass Spectrometer. *Energy Fuels* **2019,** *33* (11), 10861-10867.
4. DeBlase, A. F.; Bruening, C. R.; Lewis, W. K.; Bunker, C. E. Distinguishing Condensed-Phase Cracking Products with the Same Mass-to-Charge Ratio Using a Triple Quadrupole Mass Spectrometer in Product Scan Mode. *Energy Fuels* **2021,** *35* (16), 13030-13038.
5. Rao, P.; Kunzru, D. Thermal Cracking of JP-10: Kinetics and Product Distribution. *J. Anal. Appl. Pyrolysis* **2006,** *76* (1-2), 154-160.
6. Liu, M.; Dana, A.; Johnson, M.; Goldman, M.; Jocher, A.; Payne, A.; Grambow, C.; Han, K.; Yee, N.; Mazeau, E.; Blondal, K.; West, R.; Goldsmith, C.; Green, W. Reaction Mechanism Generator v3.0: Advances in Automatic Mechanism Generation. *J. Chem Inf. Model.* **2021,** *61* (6), 2686-2696.
7. Li, D.; Zhao, Y. Understanding the Chain Mechanism of Radical Reactions in n-Hexane Pyrolysis. *Res. Chem. Intermed.* **2015,** *41* (6), 3507-3529.
